# A novel class of antimicrobial drugs selectively targets a *Mycobacterium tuberculosis* PE-PGRS protein

**DOI:** 10.1371/journal.pbio.3001648

**Published:** 2022-05-31

**Authors:** Hoonhee Seo, Sukyung Kim, Hafij Al Mahmud, Md Imtiazul Islam, Youjin Yoon, Hyun-Deuk Cho, Kung-Woo Nam, Jiwon Choi, Young Sig Gil, Byung-Eui Lee, Ho-Yeon Song

**Affiliations:** 1 Department of Microbiology and Immunology, School of Medicine, Soonchunhyang University, Dongnam-gu, Cheonan-si, Chungnam, Republic of Korea; 2 Probiotics Microbiome Convergence Center, Soonchunhyang University, Sinchang-myeon, Asan-si, Chungnam, Republic of Korea; 3 Department of Pathology, School of Medicine, Soonchunhyang University, Dongnam-gu, Cheonan-si, Chungnam, Republic of Korea; 4 Department of Life Science and Biotechnology, School of Life Sciences, Soonchunhyang University, Sinchang-myeon, Asan-si, Chungnam, Republic of Korea; 5 College of Pharmacy, Dongduk Women’s University, Seongbuk-gu, Seoul, Republic of Korea; 6 R&D Center, Kolmarpharma Co., Ltd., Jecheon-si, Chungbuk, Republic of Korea; Brigham and Women’s Hospital, UNITED STATES

## Abstract

The continued spread of drug-resistant tuberculosis is one of the most pressing and complex challenges facing tuberculosis management worldwide. Therefore, developing a new class of drugs is necessary and urgently needed to cope with the increasing threat of drug-resistant tuberculosis. This study aims to discover a potential new class of tuberculosis drug candidates different from existing tuberculosis drugs. By screening a library of compounds, methyl (S)-1-((3-alkoxy-6,7-dimethoxyphenanthren-9-yl)methyl)-5-oxopyrrolidine-2-carboxylate (PP) derivatives with antitubercular activity were discovered. MIC ranges for PP1S, PP2S, and PP3S against clinically isolated drug-resistant *Mycobacterium tuberculosis* strains were 0.78 to 3.13, 0.19 to 1.56, and 0.78 to 6.25 μg/ml, respectively. PPs demonstrated antitubercular activities in macrophage and tuberculosis mouse models, showing no detectable toxicity in all assays tested. PPs specifically inhibited *M*. *tuberculosis* without significantly changing the intestinal microbiome in mice. Mutants selected in vitro suggest that the drug targets the PE-PGRS57, which has been found only in the genomes of the *M*. *tuberculosis* complex, highlighting the specificity and safety potency of this compound. As PPs show an excellent safety profile and highly selective toxicity specific to *M*. *tuberculosis*, PPs are considered a promising new candidate for the treatment of drug-resistant tuberculosis while maintaining microbiome homeostasis.

## Abstract 2

This study identifies a new class of PP derivatives as Mycobacterium tuberculosis-targeting antimicrobials with microbiome-safe properties that do not disrupt normal flora, making them a promising candidate for the treatment of drug-resistant tuberculosis.

## Introduction

Although it has been nearly 70 years since the first clinical trial of isoniazid (INH) for human tuberculosis [1], this disease remains a significant health concern worldwide. It is one of the leading causes of death from infectious diseases, ranking even above AIDS [2]. One-third of the world’s population (i.e., approximately 2 to 3 billion people) are infected with *Mycobacterium tuberculosis*, and approximately 7 million people are treated for active tuberculosis, with 1.5 million deaths annually [3]. The rise in people with AIDS worldwide has further complicated this issue [4]. To make matters worse, the emergence of multidrug-resistant (MDR), extensively drug-resistant (XDR), and now totally drug-resistant (TDR) strains of *M*. *tuberculosis* necessitates the development of new drugs to control this epidemic more urgently than ever [5].

Drugs used in front-line chemotherapy for drug-sensitive tuberculosis were developed more than half a century ago, primarily completed in the 1970s, with clinical studies that defined optimal combinations and durations [6]. The following 30 years were a hiatus for tuberculosis drug research and development [7,8]. This hiatus made the fight against this tuberculosis-causing organism extremely difficult [9]. In recent years, with the efforts of researchers in academia and industry, the tuberculosis drug discovery and development pipeline has continued to grow [10]. Although new compounds are progressing into the clinical development pipeline, the global drug pipeline for tuberculosis is insufficient to address unmet treatment needs [11]. Therefore, there is an urgent need to develop new tuberculosis drugs with improved antitubercular activities against drug-resistant *M*. *tuberculosis*.

We initiated a program to screen potential antitubercular compounds of natural origin with new structures and mechanisms capable of inhibiting drug-resistant strains of *M*. *tuberculosis*. After an extensive screening of plant extracts, we identified deoxypergularinine (DPG) purified from the root of *Cynanchum atratum* Bunge as a promising candidate [12]. DPG showed potential antitubercular activities against *M*. *tuberculosis* with MIC values ranging from 6.25 to 12.5 μg/ml in vitro and a CC_50_ of 18.7 μg/ml in the A549 cell line [12]. To extend our research on DPG as a new class of antituberculosis agents, we designed a combinatorial library based on 6, 7-dimethoxy-3-alkoxy-9-phenanthrenecarboxylic acid and α-[(4-alkoxyphenyl) methylene]-3,4-dimethoxybenzeneacetic acid structures of DPG. Through in vitro antitubercular activity and cytotoxicity tests for these synthesized compounds, methyl (S)-1-((3-alkoxy-6,7-dimethoxyphenanthren-9-yl)methyl)-5-oxopyrrolidine-2-carboxylate (PP) derivatives with excellent antitubercular effects and low toxicities were discovered. In the present study, we identified and characterized PPs as novel antitubercular drug candidates.

## Results

### In vitro antitubercular activities of PPs

As a result of the resazurin microtiter assay for 11 types of DPG analogs, DPGA1 (PP1S) had the lowest MIC and showed no cytotoxicity within the tested concentration range (S1 Table). Next, derivatives of PP1S were synthesized, and further in vitro evaluation was performed on them.

**Fig 1:**
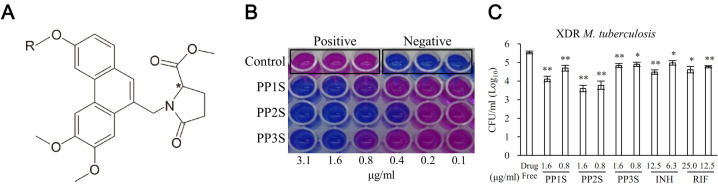
Structures of PPs and their inhibitory activities against *M*. *tuberculosis*. (A) PP derivatives are composed of PP1S, PP2S, and PP3S in which the alkoxy R group has a methyl group, a butyl group, and a benzyl group, respectively. (B) Inhibitory effect of S-form PPs on *M*. *tuberculosis* H37Rv were evaluated by resazurin microtiter assay. (C) Antitubercular effects of PPs on XDR *M*. *tuberculosis* (KMRC 00203–00197) were confirmed through CFU enumeration assay. Data are presented as mean ± SD. Student *t* test was used to compare groups (*p < 0.05, **p < 0.01). CFU, colony-forming units; PP, methyl (S)-1-((3-alkoxy-6,7-dimethoxyphenanthren-9-yl)methyl)-5-oxopyrrolidine-2-carboxylate; XDR, extensively drug-resistant. Data underlying this Figure can be found in S1 Data.

Structures of PPs and their in vitro antitubercular effects are shown in Fig 1. PPs are composed of PP1S, PP2S, and PP3S, in which the alkoxy R group has methyl, butyl, and benzyl group, respectively (Fig 1A). PPs have a chiral center. With an S-form, PP1S, PP2S, and PP3S had in vitro antitubercular activities, whereas PP1R, PP2R, and PP3R with an R-form did not (S2 Table). Testing antitubercular effects of PPs against *M*. *tuberculosis* H37Rv with the resazurin microtiter assay showed that MICs of PP1S and PP3S were the same at 1.6 μg/ml, while the MIC of PP2S was lower at 0.4 μg/ml (Fig 1B). Effects of PPs on XDR *M*. *tuberculosis* were confirmed through colony-forming unit (CFU) enumeration assay (Fig 1C). PPs at both 0.8 μg/ml and 1.6 μg/ml showed significant effects. At 0.8 μg/ml, PP1S, PP2S, and PP3S showed log reductions of 0.8, 1.8, and 0.5, respectively. At 1.6 μg/ml, they showed log reductions of 1.3, 1.9, and 0.6, respectively. However, INH and rifampicin (RIF) showed log reductions of 1.0 and 0.6, respectively, at 12.5 μg/ml, a much higher concentration than PPs.

**Fig 2:**
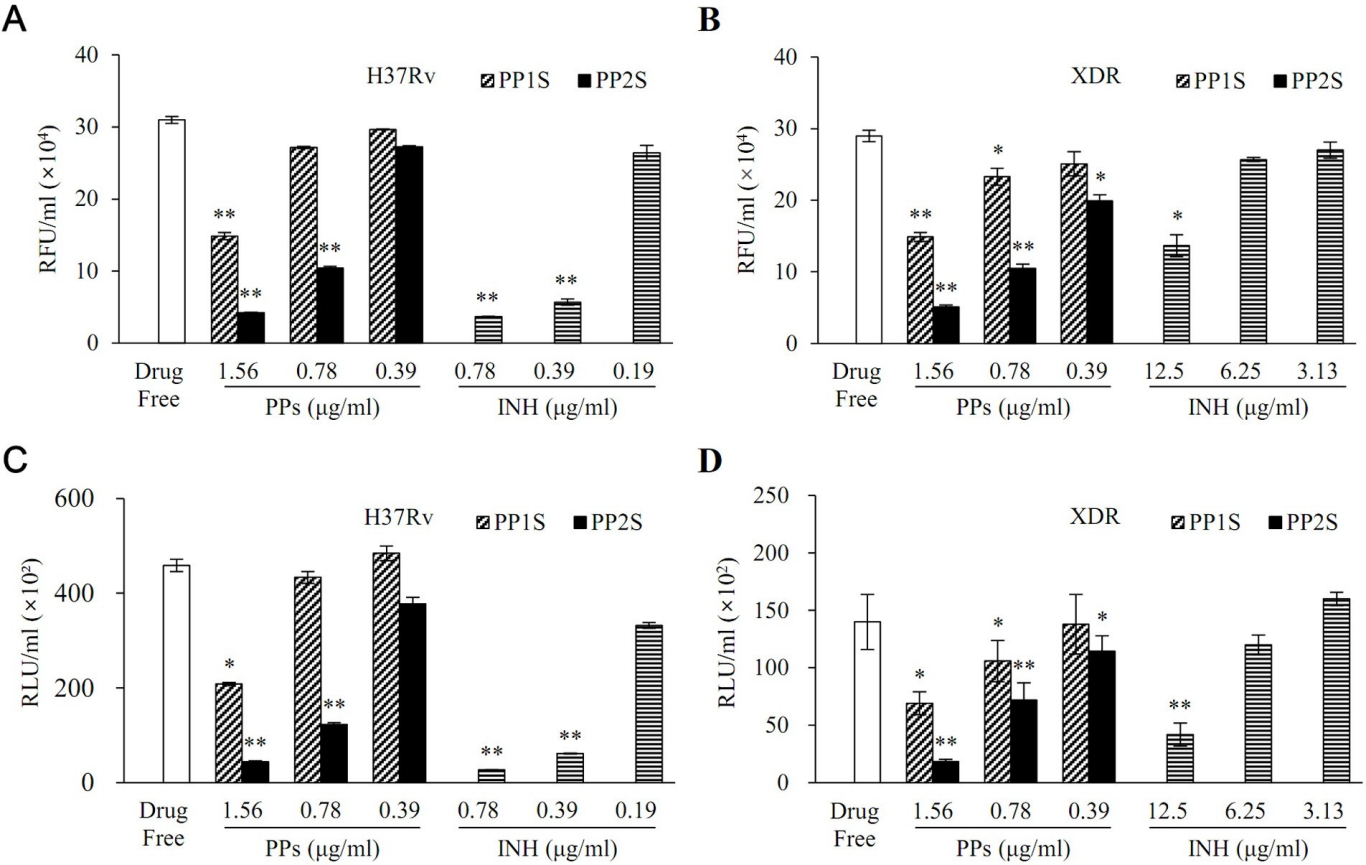
In vitro antitubercular activities of PPs against (A and C) *M*. *tuberculosis* H37Rv and (B and D) XDR *M*. *tuberculosis* measured by (A and B) fluorescence-based resazurin microtiter assay and (C and D) luminescence-based microbial cell viability assay. Experiments were performed in triplicate and repeated 3 times. Data are presented as mean ± SD and Student *t* test was used to compare groups (*p < 0.05, **p < 0.01). INH, isoniazid; PP, methyl (S)-1-((3-alkoxy-6,7-dimethoxyphenanthren-9-yl)methyl)-5-oxopyrrolidine-2-carboxylate; XDR, extensively drug-resistant. Data underlying this Figure can be found in S1 Data.

Antitubercular activities of PPs were further evaluated with fluorescence and luminescence-based assay (Fig 2). For *M*. *tuberculosis* H37Rv, both methods showed significant antitubercular effects of PP1S at 1.56 μg/ml and PP2S at 1.56 and 0.78 μg/ml (Fig 2A and 2C). INH used as a control showed a significant antitubercular effect at lower concentrations of 0.78 μg/ml and 0.39 μg/ml. For XDR *M*. *tuberculosis*, with both methods, PP1S showed a significant antitubercular effect at 1.56 and 0.78 μg/ml, while PP2S showed a significant inhibitory effect at a lower concentration of 0.39 μg/ml (Fig 2B and 2D). However, INH used as a control did not show a significant antitubercular effect at a higher concentration of 6.25 or 3.13 μg/ml.

### Activities of PPs against clinically isolated MDR, XDR, and TDR strains of *M*. *tuberculosis*

Using the resazurin microtiter assay, antitubercular activities of PPs against MDR, XDR, and TDR *M*. *tuberculosis* clinical isolates were also evaluated (Table 1). MIC values of PP1S, PP2S, and PP3S were 0.4 to 6.3 μg/ml, 0.2 to 1.6 μg/ml, and 0.8 to 6.3 μg/ml, respectively, which were lower than those of existing first-line antitubercular drugs.

**Table 1:** In vitro antitubercular activity of PPs and control drugs against clinically isolated MDR, XDR, and TDR *M*. *tuberculosis* strains.

	MIC (μg/ml)^a^							
*M*. *tuberculosis*	INH	RIF	STR	PZA	EMB	PP1S	PP2S	PP3S
MDR-1	12.5	>100	>100	>100	6.3	0.8	0.4	3.1
MDR-2	12.5	>100	100	>100	6.3	1.6	0.8	6.3
MDR-3	12.5	>100	1.6	>100	6.3	1.6	0.8	3.1
MDR-4	3.1	>100	0.4	>100	3.1	0.8	0.8	3.1
MDR-5	25	>100	0.8	>100	6.3	1.6	0.8	6.3
MDR-6	12.5	3.1	3.1	>100	6.3	0.8	0.8	1.6
MDR-7	12.5	3.1	3.1	>100	6.3	0.8	0.8	3.1
MDR-8	50	>100	>100	>100	6.3	3.1	1.6	6.3
MDR-9	12.5	>100	3.13	>100	6.3	1.6	0.8	3.1
MDR-10	25	>100	25	>100	3.1	0.8	0.8	3.1
MDR-11	3.1	3.1	>100	>100	0.4	0.8	0.8	3.1
XDR-1	25	100	0.8	>100	6.3	1.6	0.8	6.3
XDR-2	12.5	>100	0.8	>100	6.3	0.8	0.4	3.1
XDR-3	3.1	>100	50	>100	6.3	6.3	0.4	3.1
XDR-4	3.1	>100	>100	>100	6.3	0.4	0.4	1.6
XDR-5	12.5	6.3	0.8	>100	6.3	0.8	0.8	3.1
XDR-6	12.5	>100	50	>100	6.3	0.8	0.4	6.3
XDR-7	6.3	>100	0.8	>100	12.5	1.6	0.8	6.3
XDR-8	12.5	>100	>100	>100	6.3	0.8	0.4	6.3
XDR-9	50	>100	>100	>100	6.3	0.4	0.8	6.3
TDR-1	12.5	>100	50	>100	6.3	3.3	1.6	0.8
TDR-2	6.3	>100	>100	>100	6.3	0.8	0.2	1.6
TDR-3	12.5	>100	50	>100	6.3	3.1	1.6	0.8

^a^Determined by resazurin microtiter assay.

INH, isoniazid; MDR, multidrug-resistant *M*. *tuberculosis*; PP, methyl (S)-1-((3-alkoxy-6,7-dimethoxyphenanthren-9-yl)methyl)-5-oxopyrrolidine-2-carboxylate; RIF, rifampicin; TDR, totally drug-resistant *M*. *tuberculosis*; XDR, extensively drug-resistant *M*. *tuberculosis*.

### Intracellular killing activities of PPs against *M*. *tuberculosis*

**Fig 3:**
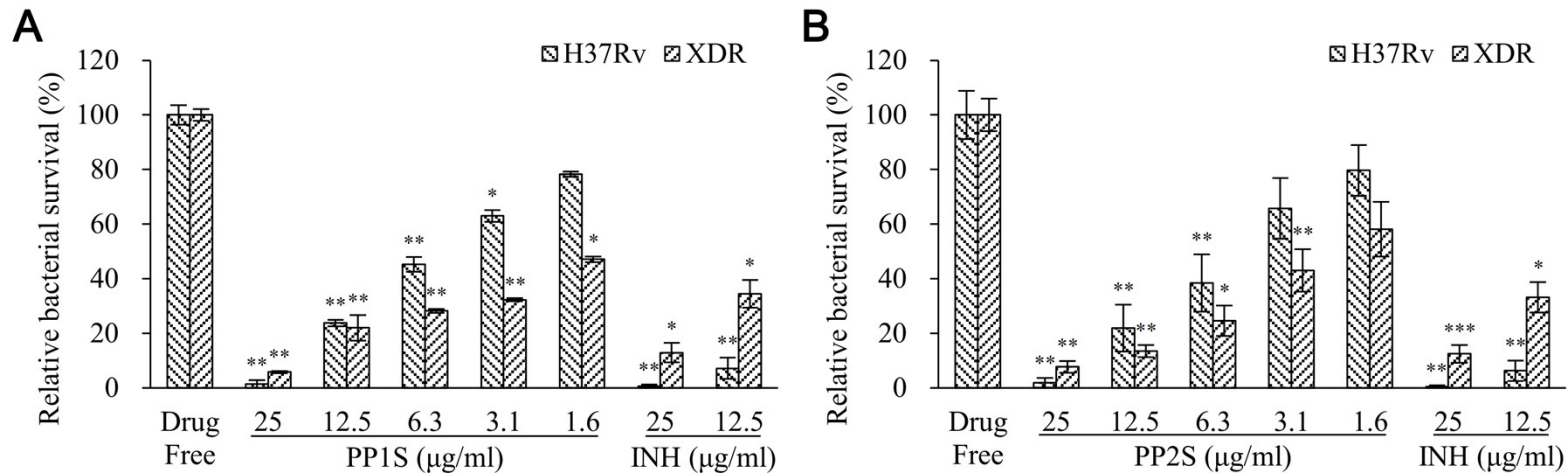
Intracellular killing activities of PPs for *M*. *tuberculosis*. Inhibitory effects of (A) PP1S and (B) PP2S on *M*. *tuberculosis* H37Rv and XDR *M*. *tuberculosis* infected in macrophages were analyzed through an intracellular antimycobacterial activity test. (A and B) Survival of *M*. *tuberculosis* in macrophages was quantified by a CFU assay. Experiments were performed in triplicate and repeated 3 times. Data are presented as mean ± SD and Student *t* test was used to compare groups (*p < 0.05, **p < 0.01). CFU, colony-forming units; INH, isoniazid; PP, methyl (S)-1-((3-alkoxy-6,7-dimethoxyphenanthren-9-yl)methyl)-5-oxopyrrolidine-2-carboxylate; XDR, extensively drug-resistant. Data underlying this Figure can be found in S1 Data.

Antitubercular efficacies of PPs in Raw 264.7 cells, a macrophage cell line, were determined (Fig 3). Results of the CFU test revealed that PP1S and PP2S significantly inhibited the growth of *M*. *tuberculosis* H37Rv and XDR *M*. *tuberculosis* within the concentration range tested in a concentration-dependent manner (Fig 3A and 3B). Treatment with 1 μg/ml of PP2S in cells infected with green fluorescent protein (GFP)-expressing *M*. *tuberculosis* led to a decrease in green signal, confirming the bactericidal activity of PP2s (S2 Fig).

### In vivo efficacy in a mouse model of pulmonary tuberculosis

**Fig 4:**
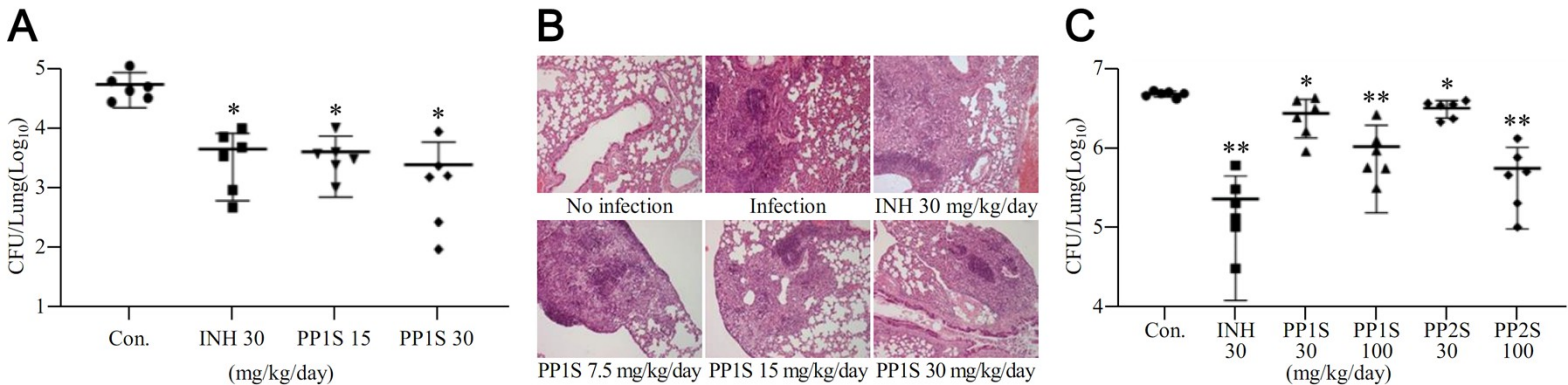
Efficacies of PPs in a BALB/C mouse model of pulmonary tuberculosis. After 4 weeks of low-dose infection followed by 4 weeks of oral administration of PP1S, (A) bacterial load in the lung was measured through CFU test, and (B) granuloma area was analyzed after HE staining (magnification, ×100). (C) After 10 days of high-dose infection followed by oral administration of PP1S or PP2S for 2 weeks, bacterial load in the lung was measured through the CFU test. INH was used as a positive control at 30 mg per kg body weight. Data are expressed as mean ± SD and Student *t* test was used to compare groups (*p < 0.01, **p < 0.001). Images of Ziehl–Neelsen staining in a high-dose infection model are presented in Supporting information (S3 Fig). CFU, colony-forming units; HE, hematoxylin–eosin; INH, isoniazid; PP, methyl (S)-1-((3-alkoxy-6,7-dimethoxyphenanthren-9-yl)methyl)-5-oxopyrrolidine-2-carboxylate. Data underlying this Figure can be found in S1 Data.

Antitubercular efficacies of PPs were then evaluated in a mouse model of tuberculosis (Fig 4). After 4 weeks of low-dose infection, 4 weeks of administration of PP1S at 30 mg/kg reduced bacterial load in lungs by more than 90% compared to the drug-free group (Fig 4A). This effect was similar to the administration of INH at 30 mg/kg to mice under the same conditions. In the control group, mouse lung sections contained multiple large, confluent granulomatous foci, whereas a limited number of small granulomatous lesions were found in the lungs of mice treated with PP1S or INH (Fig 4B). An initial bactericidal efficacy trial was performed using BALB/c mice infected with a high and ultimately lethal inoculum of *M*. *tuberculosis*. Bacterial burden in the lungs of mice was significantly reduced after 10 days of PP2S treatment compared to that in the control group (Fig 4C).

### Cytotoxicities of PPs to human and murine cells

Cytotoxicities of PPs to human and murine cells were evaluated (Table 2). Like other first-line drugs, PP1S and PP2S did not show any cytotoxicity to human cells A549, HEK-293, THP-1, or SH-SY5Y or murine cells Raw264.7 or L929 tested. For PP3S, it was not cytotoxic to A549 or THP-1 human cells or murine cells Raw264.7 or L929 tested. However, its CC_50_ was 7.8 μg/ml for human cells HEK-293 and SH-SY5Y.

**Table 2:** CC_50_ values of PPs against 6 different types of cells.

	CC_50_ (μg/ml)					
	Human cells				Murine cells	
Drugs	A549	HEK-293	THP-1	SH-SY5Y	Raw264.7	L929
INH	>500	>500	>500	>500	>500	>500
RIF	>500	>500	>500	>500	>500	>500
PP1S	>500	>500	>500	>500	>500	>500
PP2S	>500	>500	>500	>500	>500	>500
PP3S	>500	7.8	>500	7.8	>500	>500
DNF-3^a^	12.5	12.5	12.5	12.5	12.5	12.5

^a^DNF-3 [45] with antitubercular activity was also tested as a positive control.

INH, isoniazid; RIF, rifampicin.

### Single/repeated oral toxicity, genotoxicity toxicity, and pharmacokinetic studies

**Fig 5:**
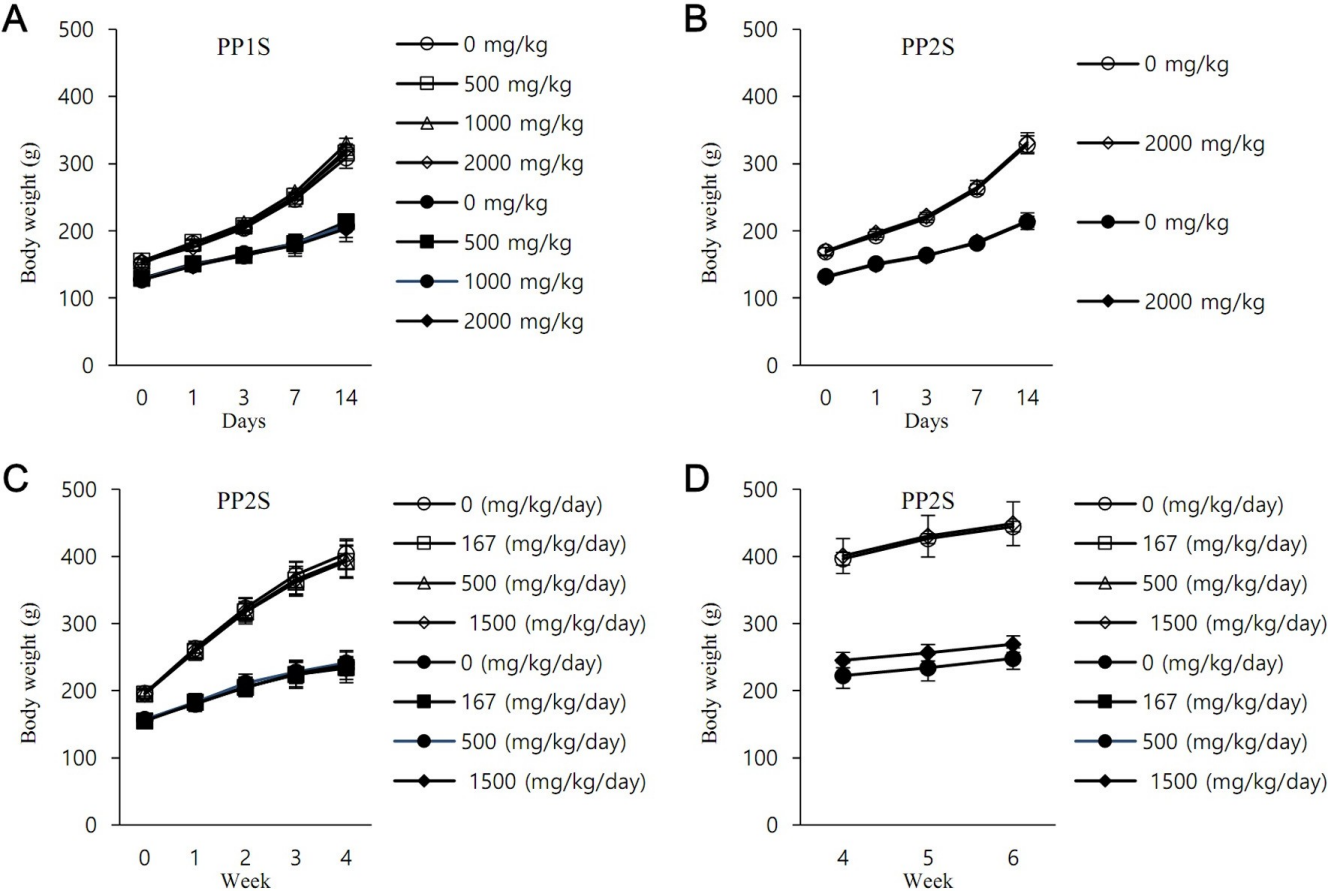
Body weights of SD rats after single or repeated oral administration of PPs. Bodyweight was measured for 2 weeks after a single administration of (A) PP1S or (B) PP2S. (C) PP2S was repeatedly administered for 4 weeks, and body weight was measured. (D) After the dosing period, the control and maximum administration groups had a recovery period of 2 weeks, and their body weight was continuously measured. Colored symbols indicate females and uncolored symbols indicate males. PP, methyl (S)-1-((3-alkoxy-6,7-dimethoxyphenanthren-9-yl)methyl)-5-oxopyrrolidine-2-carboxylate; SD, Sprague Dawley. Data underlying this Figure can be found in S1 Data.

Single and repeated oral toxicity studies of PPs were performed using Sprague Dawley (SD) rats (Fig 5). After oral administration of PP1S or PP2S at a dose up to 2,000 mg/kg, clinical symptoms and death were not observed for 2 weeks in any group. There was no significant change in body weight compared to the control group (Fig 5A and 5B). In the 4-week repeated oral toxicity study, PP2S at a dose up to 1,500 mg/kg/day (4 weeks of administration and 2 weeks of recovery) did not cause death or abnormal clinical symptoms. It did not lead to a significant change in body weight compared to the control group (Fig 5C and 5D) and no organ weight/histopathological changes were observed (S3 and S4 Tables). Moreover, PP2S did not show genotoxicity in bacterial reverse mutation test (S5 Table), chromosomal aberration test (S6 Table), or bone marrow micronucleus formation test (S7 Table). The toxicity of PP2S after a single oral administration to mice is presented in S8 Table. Results of a pharmacokinetic study using female rats after a single oral administration of PP2S presented in S4 Fig.

### Antimycobacterial activities of PPs against *Mycobacterium* species

An antimycobacterial activity test using a resazurin microtiter assay was performed on various species belonging to the clinically isolated *Mycobacterium* genus (Table 3). INH, RIF, streptomycin (STR), and ethambutol (EMB) inhibited the growth of most species, but PPs had no effect except for on *Mycobacterium bovis*.

**Table 3:** MIC values of PPs for species belonging to *Mycobacterium* genus.

	MIC (μg/ml)^a^							
Species	PP1S	PP2S	PP3S	INH	RIF	STR	EMB	PZA
*M*. *abscessus*	>100	>100	>100	>100	>100	25	100	>100
*M*. *arupense*	>100	>100	>100	50	0.1	0.1	0.4	>100
*M*. *aubagnense*	>100	>100	>100	>100	1.6	>100	50	>100
*M*. *avium*	>100	>100	>100	25	1.6	3.1	50	>100
*M*. *bolletii*	>100	>100	>100	>100	>100	25	>100	>100
*M*. *bovis*	1.6	0.8	3.12	0.2	0.1	0.4	1.6	>100
*M*. *chitae*	>100	>100	>100	12.5	6.3	>100	0.2	>100
*M*. *colombiense*	>100	>100	>100	12.5	0.2	0.8	6.3	>100
*M*. *conceptionense*	>100	>100	>100	25	25	3.1	50	>100
*M*. *fortuitum*	>100	>100	>100	25	0.2	6.3	12.5	>100
*M*. *gilvum*	>100	>100	>100	12.5	0.2	0.2	12.5	>100
*M*. *goodie*	>100	>100	>100	25	100	0.2	0.8	>100
*M*. *gordonae*	>100	>100	>100	>100	3.1	100	>100	>100
*M*. *heraklionease*	>100	>100	>100	>100	0.2	50	100	>100
*M*. *intracellulare*	>100	>100	>100	25	<0.1	0.8	3.1	>100
*M*. *kansasii*	>100	>100	>100	0.2	<0.1	0.2	1.6	>100
*M*. *kyorinense*	>100	>100	>100	3.1	50	0.8	50	>100
*M*. *marinum*	>100	>100	>100	25	<0.1	<0.1	3.1	>100
*M*. *marseiliense*	>100	>100	>100	>100	>100	25	100	>100
*M*. *massiliense*	>100	>100	>100	12.5	0.2	0.8	100	>100
*M*. *neoaurum*	>100	>100	>100	3.1	0.2	0.8	6.3	>100
*M*. *peregrinum*	>100	>100	>100	6.3	25	3.1	3.1	>100
*M*. *phlei*	>100	>100	>100	>100	25	0.2	1.6	>100
*M*. *phocaicum*	>100	>100	>100	>100	25	6.3	0.4	>100
*M*. *smegmatis*	>100	>100	>100	25	25	0.2	0.8	>100
*M*. *szulgai*	>100	>100	>100	1.6	<0.1	0.2	3.1	>100
*M*. *xenopi*	>100	>100	>100	1.6	0.2	0.2	25	>100

^a^Determined by resazurin microtiter assay.

EMB, ethambutol; INH, isoniazid; PZA, pyrazinamide; RIF, rifampicin; STR, streptomycin.

### Antibacterial activities of PPs against clinically significant nonmycobacterial microorganisms

PPs were further tested for their in vitro antimicrobial activities against clinically significant microorganisms (17 gram-negative bacteria, 6 gram-positive bacteria, and 7 fungal strains) (Table 4). Results showed that RIF and STR broadly inhibited bacterial growth. However, similar to INH, PPs did not inhibit the growth of 30 pathogens tested.

**Table 4:** MIC values of PPs and control drugs against 23 gram-positive/gram-negative bacteria and 7 fungal strains.

		MIC (μg/ml)^a^					
	Microorganisms	PP1S	PP2S	PP3S	INH	RIF	STR
Gram-negative	*Acinetobacter baumannii*	>100	>100	>100	>100	3.1	6.3
	*Citrobacter freundii*	>100	>100	>100	>100	25	1.6
	*Enterobacter aerogenes*	>100	>100	>100	>100	6.3	3.1
	*Escherichia coli*	>100	>100	>100	>100	6.3	25
	*Escherichia coli* O157	>100	>100	>100	>100	3.1	6.3
	*Klebsiella pneumoniae*	>100	>100	>100	>100	6.3	3.1
	*Proteus mirabilis*	>100	>100	>100	>100	3.1	6.3
	*Proteus vulgaris*	>100	>100	>100	>100	6.3	25
	*Pseudomonas aeruginosa*	>100	>100	>100	>100	25	25
	*Salmonella Enteritidis*	>100	>100	>100	>100	12.5	3.1
	*Salmonella Paratyphi* A	>100	>100	>100	>100	12.5	6.3
	*Salmonella Typhimurium*	>100	>100	>100	>100	12.5	25
	*Serratia marcescens*	>100	>100	>100	>100	12.5	50
	*Shigella boydii*	>100	>100	>100	>100	0.4	25
	*Shigella dysenteriae*	>100	>100	>100	>100	1.6	25
	*Shigella flexneri*	>100	>100	>100	>100	1.5	25
	*Shigella sonnei*	>100	>100	>100	>100	3.1	3.1
Gram-positive	*Bacillus subtilis*	>100	>100	>100	>100	<0.1	0.8
	*Corynebacterium diphtheriae*	>100	>100	>100	>100	0.05	3.1
	*Staphylococcus aureus*, MSSA	>100	>100	>100	>100	0.05	6.3
	*Staphylococcus aureus*, MRSA	>100	>100	>100	>100	0.05	3.1
	*Staphylococcus epidermidis*	>100	>100	>100	>100	0.05	1.6
	*Streptococcus pneumoniae*	>100	>100	>100	>100	0.05	12.5
Fungi	*Aspergillus fumigatus*	>100	>100	>100	>100	>100	>100
	*Candida albicans*	>100	>100	>100	>100	>100	>100
	*Cryptococcus neoformans*	>100	>100	>100	>100	>100	>100
	*Microsporum canis*	>100	>100	>100	>100	>100	>100
	*Rhizopus oryzae*	>100	>100	>100	>100	>100	>100
	*Saccharomyces cerevisiae*	>100	>100	>100	>100	>100	>100
	*Trichophyton rubrum*	>100	>100	>100	>100	>100	>100

INH, isoniazid; MRSA, methicillin-resistant *Staphylococcus aureus*; MSSA, methicillin-susceptible *Staphylococcus aureus*; RIF, rifampicin; STR, streptomycin.

^a^In vitro MIC values were determined by broth dilution procedures described by the Clinical and Laboratory Standards Institute (CLSI).

### Effect of PP on the gut microbiome in mice

**Fig 6:**
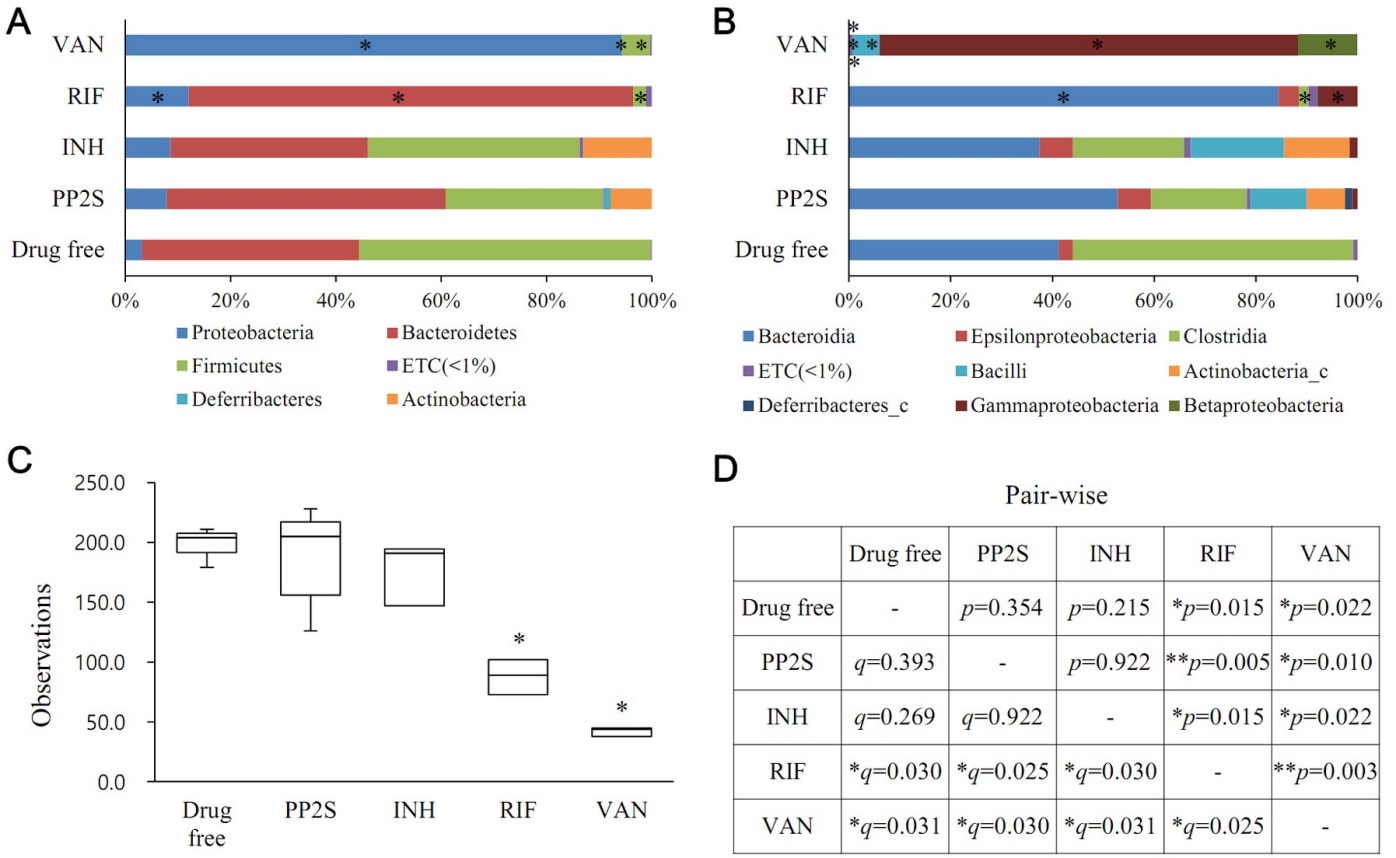
Gut microbiota changes after drug treatment. (A and B) Effects of drugs on intestinal microbiome were investigated through 16s rRNA gene-based metagenomics analysis. Compared to the drug-free control group, the microbiome distribution was significantly changed in the group administered with RIF or VAN but did not show any significant change in the group treated with PP2S or INH (*p < 0.05). (C) A phylogenetic diversity analysis to investigate the biodiversity of intestinal microbiome upon administration of each drug (*p < 0.05). (D) A beta set-significance analysis was performed using permutational multivariate analysis of variance (PERMANOVA) based on Jensen–Shannon distances to show associations of gut microbiomes between groups (*p < 0.05, **p < 0.01, *q < 0.05). INH, isoniazid; RIF, rifampicin; VAN, vancomycin. Data underlying this Figure can be found in S1 Data.

To investigate the drug’s effect on the intestinal microbiome, each drug was administered orally at 30 mg/kg once a day for 7 days using BALB/c mice. Feces were collected for 16s rRNA gene-based metagenomics analysis (Fig 6). The PP2S-treated or INH-treated groups did not significantly differ in phylum or class level for all taxa compared to the drug-free group. However, there were significant differences between groups in Proteobacteria, Bacteroidetes, and Firmicutes phyla and Bacteroidia, Clostridia, and Gammaproteobacteria classes in RIF and vancomycin (VAN) treatment groups. In the VAN-treated group, there were also significant differences between groups in Epsilonproteobacteria, Bacilli, and Betaproteobacteria classes (Fig 6A and 6B). In terms of phylogenetic diversity, there was no significant change in the group treated with PP2S or INH. However, phylogenetic diversity was significantly decreased in the group treated with RIF or VAN than in the drug-free control group (Fig 6C). In beta set-significance analysis, pair-wise comparison between the drug-free group and the PP2S-treated or INH-treated groups was not significant. However, differences in gut microbiome between RIF or VAN and other groups were significant (Fig 6D).

### Isolation of PPs-resistant strains and estimation of drug target

**Fig 7:**
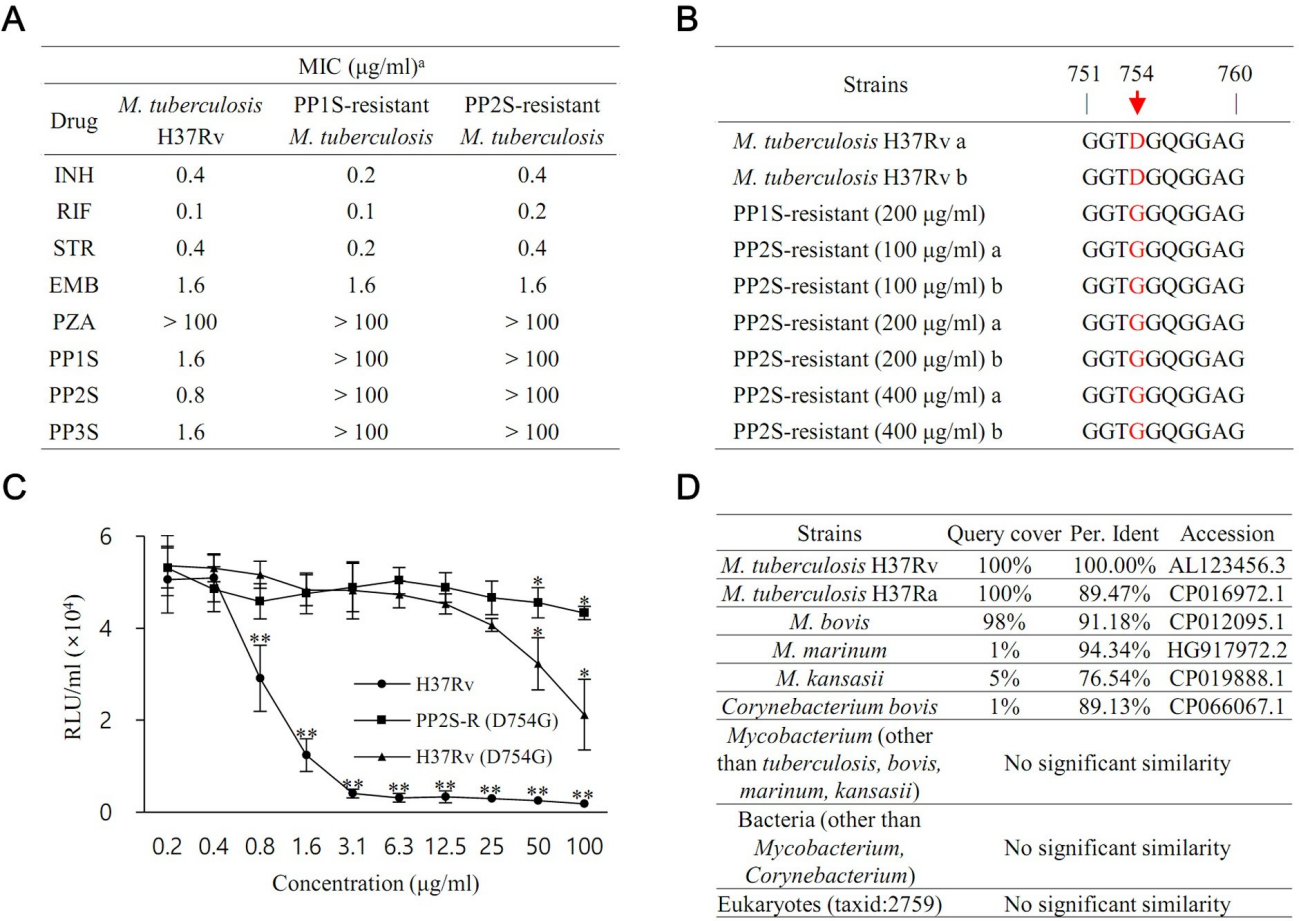
Identification of PPs target. (A) PPs-resistant *M*. *tuberculosis* strains were prepared, and resistance was verified by a resazurin microtiter assay. (B) As a result of whole-genome sequencing analysis of resistant strains, it was confirmed that the sequence encoding the 754th amino acid of *Rv3514* was commonly mutated. (C) It was confirmed that resistance to PPs occurs when the H37Rv strain is artificially induced through point mutagenesis. (D) As a result of NCBI Nucleotide BLAST analysis of the sequence of *Rv3514*, it was confirmed that the similarity was low or absent except for the *M*. *tuberculosis* complex. Data are expressed as mean ± SD. Statistical significance with initial value was analyzed using unpaired Student *t* test. *, *p* < 0.05; **, *p* < 0.01. BLAST, Basic Local Alignment Search Tool; EMB, ethambutol; INH, isoniazid; NCBI, National Center for Biotechnology Information; PP, methyl (S)-1-((3-alkoxy-6,7-dimethoxyphenanthren-9-yl)methyl)-5-oxopyrrolidine-2-carboxylate; PZA, pyrazinamide; RIF, rifampicin; STR, streptomycin. Data underlying this Figure can be found in S1 Data.

To gain insight into the molecular target of PP, random mutants spontaneously resistant to PPs were prepared (Fig 7). At a concentration of 100 μg/ml for PP2S, resistant strains were generated with a frequency of about 10^‒7^ (S5 Fig). At 200 μg/ml and above, resistant strains did not occur directly. However, strains resistant to high concentrations of PPs were prepared by transferring and reinoculating resistant strains generated at 100 μg/ml. The mutants showed a consistent increase in MIC for PPs by several orders of magnitude but remained susceptible to first-line drugs (Fig 7A). Sequence alignment showed that the only gene commonly affected in all 7 independent mutants encodes PE-PGRS57 (*Rv3514*) (Fig 7B). Sequence analysis confirmed that the mutation of Asp to Gly was associated with resistance to PPs. For more in-depth verification, a mutation was induced from Asp to Gly at the 754th amino acid of PE_PGRS57 for H37Rv using point mutagenesis (Fig 7C). As a result of the antitubercular test against H37Rv (D754G), PPs up to 50 μg/ml showed resistance.

As a result of BLAST (Basic Local Alignment Search Tool) analysis by the NCBI (National Center for Biotechnology Information) for the nucleotide sequence of *Rv3514*, it was confirmed that the similarity was low or absent, except for the *M*. *tuberculosis* complex of *M*. *tuberculosis* and *M*. *bovis* (Fig 7D). Antimycobacterial test results for various *M*. *bovis* bacillus Calmette–Guérin (BCG) strains are presented in S9 Table. This sequence similarity analysis result is consistent with the test mentioned above for humans, mice, gram-positive/gram-negative bacteria, fungi, and *Mycobacterium*.

## Discussion

As a pathogenic organism, *M*. *tuberculosis* continues to evolve resistance to frontline drugs, leading to the emergence of MDR, XDR, and ultimately TDR *M*. *tuberculosis* [13]. Accordingly, the development of a new class of antituberculosis agents is urgently needed. Here, we report antituberculosis effects of PPs, a new class of drugs utterly different from existing tuberculosis medications structurally. PPs are derivatives of DPGA1, one of the DPG analogues (DPGA) discovered during the study to improve the antitubercular activity and toxicity issue of DPG (S1 Supporting Chemistry Schemes). In terms of structure–activity relationship (SAR), when the hexahydro-indolizine structure of DPG was substituted with methyl-5-oxopyrrolidine-2-carboxylate, antituberculous activity was increased but the toxicity was lowered. On the other hand, substitution with a methoxycarbonyl group or a hydroxymethyl group did not increase its antitubercular activity. It has been confirmed that the cleaved form of phenanthrene compounds have antitubercular activities, and it is worth conducting further studies. Among PPs having 3 alkoxy groups, PP2S, in which the alkoxy substituent is a butyl group, showed a better in vitro antitubercular effect. From this observation, it was clear that the substitution of the alkoxy group on carbon 3 of the phenanthromethyl group was related to the antitubercular effect. Unlike S-form, R-form had no antitubercular effect, confirming that the chirality of PPs was also an essential factor in the antitubercular effect. In particular, PP2S has not been reported in the existing literature. Further studies on its stability in blood and activation process/activation form are needed.

Our results also demonstrate that PPs have antitubercular activities against both drug-susceptible and drug-resistant strains of *M*. *tuberculosis*. Overall, we observed similar MICs against *M*. *tuberculosis* in vitro, even for highly drug-resistant isolates. Crucially, PPs displayed similar activities against all clinical isolates of *M*. *tuberculosis* tested, including drug-resistant strains, indicating that they might target a previously unknown biological function of *M*. *tuberculosis*. However, further kinetic studies of PPs against various strains of *M*. *tuberculosis* are needed.

Infection of the host begins after inhalation of an aerosol containing a small number of bacilli [14]. Once they enter the lungs, they are internalized through phagocytosis by alveolar macrophages; thus, these bacilli could remain in the lungs or spread to other organs [14]. As such, tuberculosis is an infectious disease that can involve almost any extrapulmonary site. It is usually characterized by necrotic granulomatous inflammation in the lungs pathologically [15]. In this context, the test for inhibitory effects of PPs against *M*. *tuberculosis* was extended to a macrophage infection model. Results confirmed that the inhibitory effect on *M*. *tuberculosis* was still effective in cells. In particular, the effect was even better for drug-resistant strains. Further studies are needed to elucidate the reason. Antitubercular activity was confirmed in a mouse model of low-dose and high-dose aerosol-infected pulmonary tuberculosis. However, a more extensive animal efficacy evaluation is needed.

Given that MDR tuberculosis requires at least 18 to 24 months of treatment with 4 to 6 medications known to have toxic side effects [16], it is vital to consider the safety profile of a new drug candidate. Therefore, cytotoxicity tests, in vivo oral toxicity tests, and genotoxicity tests were performed for PPs. No detectable toxicity was found as a result of the tests. In addition to these toxicity issues, recently, awareness of microbiome dysbiosis, which inevitably occurs in tuberculosis treatment, and the induction of various diseases it causes have begun to emerge [17–19]. Therefore, a targeted antimicrobial approach is needed to suppress key pathogens while maintaining microbial homeostasis [20]. PP2S satisfies the above in that it does not inhibit general pathogens and NTMs (nontuberculosis mycobacteria), but only *M*. *tuberculosis* complex and does not cause a change in the microbiome of mouse feces.

PPs-resistant *M*. *tuberculosis* strains artificially prepared maintained sensitivity to primary tuberculosis drugs. As shown in this result, the lack of cross-resistance to currently used antitubercular drugs is closely related to the effect of PPs on drug-resistant *M*. *tuberculosis*. Moreover, the consistent missense mutation in all PPs-resistant mutants of *M*. *tuberculosis* suggests that the drug targets the PE-PGRS57. This has been verified through a point mutagenesis experiment. However, PPs might have other targets because the point-mutant strain showed sensitivity to high concentrations of PPs. Further research is needed. The function of the PE-PGRS protein family is largely unknown [21], but some evidence indicates that its members have a role in mycobacterial infection and persistence within host tissues [22] and are regarded as promising new drug targets [21]. These suggest that PE_PGRS57 is closely related to the inhibitory effect of PPs on *M*. *tuberculosis* and the highly selective toxicity of PPs.

In summary, we identified a new structural class of antitubercular drug candidates, PPs, with good safety profiles and highly selective toxicities specific for the *M*. *tuberculosis* complex. Taken together, our data suggest that PPs are promising new candidates for treating drug-resistant tuberculosis. However, more in-depth action mechanism studies, extensive animal efficacy and safety studies, and clinical trials are needed.

## Methods

### Synthesis of PPs derivatives and analogues

According to a published method [23], 3, 6, 7-trimethoxy-9-phenanthrenemethanol was synthesized using homoveratric acid and 4-methoxybenzaldehyde as starting materials (S1 Scheme). (S)-N-[(2, 3, 6-trimethoxy-10-phenanthryl)methyl]-pyroglutamic acid methyl ester (PP1S) was prepared with a reported method [24] as shown in S1 Scheme from compound 3, 6, 7-trimethoxy-9-phenanthrenemethanol in situ. Compound PP2S was prepared using the same procedures and materials as PP1S except that homoveratric acid and 4-(n-butyloxy)benzaldehyde were used as starting materials. Compound PP3S and PP3R were synthesized as previously described [25]. PP analogues were synthesized (S2 Scheme). All new compounds were characterized by ^1^H-NMR and mass spectroscopy. Their purities were measured by thin-layer chromatography (TLC) and high-performance liquid chromatography (HPLC) analysis. Enantiomers were identified with a circular dichroism detector (Chirascan plus, Applied Photophysics, United Kingdom) (S1 Fig). Full details of chemical synthesis for compounds described in this paper are shown in Supporting information (S1 Supporting Chemistry Schemes).

### *M*. *tuberculosis* strains

H37Ra (ATCC 35835) and H37Rv (ATCC 27294) strains of *M*. *tuberculosis* were purchased from the American Type Culture Collection (ATCC, Manassas, Virginia, United States of America). Clinically isolated MDR (KMRC 00116–00023, KMRC 00116–00072, KMRC 00116–00082, KMRC 00116–00087, KMRC 00116–00107, KMRC 00116–00111, KMRC 00116–00122, KMRC 00116–00150, KMRC 00116–00181, KMRC 00116–00185, KMRC 00116–00232), XDR (KMRC 00203–00197, KMRC 00203–00052, KMRC 00203–00060, KMRC 00203–00063, KMRC 00203–00085, KMRC 00203–00092, KMRC 00203–00117, KMRC 00203–00128, KMRC 00203–00169), and TDR (KMRC 00203–00005, KMRC 00203–00051, KMRC 00203–00151) strains were purchased from the Korean Microorganism Resource Center (KMRC) (Chungbuk, Korea). Clinically isolated TDR strains were found to be resistant to kanamycin (30 μg/ml), capreomycin (40 μg/ml), prothionamide (40 μg/ml), cycloserine (30 μg/ml), para-aminosalicylic acid (1 μg/ml), ofloxacin (4 μg/ml), rifabutin (20 μg/ml), moxifloxacin (2 μg/ml), amikacin (30 μg/ml), and levofloxacin (2 μg/ml) by the absolute concentration method using Lowenstein–Jensen medium.

### Commercial drugs

INH, STR, RIF, pyrazinamide (PZA), EMB, ethionamide (ETH), methicillin (METH), and VAN were purchased from Sigma-Aldrich (USA) and used for microbiological testing.

### Resazurin microtiter assay

MICs against mycobacteria were determined with the resazurin microtiter assay [26] with minor modifications. Briefly, inocula of about 2 × 10^5^ CFU/ml were prepared by diluting *M*. *tuberculosis* stock (2 × 10^8^ CFU/ml) at 1:1,000 with 7H9 broth (BD, USA) containing 10% albumin dextrose catalase (ADC, BD, USA) and 0.2% glycerol (Samchun, Korea). Subsequently, 200 μl of the inoculum containing a predetermined concentration of drugs was dispensed into 96-well microtiter plates. Following 7 days of incubation at 37°C, 20 μl of 0.02% resazurin sodium salt solution (Sigma, USA) was added to each well. Color changes were assessed after an additional 2-day incubation at 37°C. The blue-colored resazurin was reduced and turned pink as cells grew. MIC was determined as the lowest drug concentration that prevented this color change [26]. Fluorescence readings were also measured at an excitation wavelength of 570 nm and emission wavelength of 600 nm using a multilabel reader (Victor X3, Perkin Elmer, USA).

### CFU enumeration assay

CFU enumeration assay was used to measure the killing effect of a drug on XDR *M*. *tuberculosis* (KMRC 00203–00197). Briefly, *M*. *tuberculosis* (2 × 10^5^ CFU/ml) in Middlebrook 7H9 broth (BD, USA) supplemented with ADC (BD, USA) and 0.2% glycerol (Samchun, Korea) was treated with a predetermined concentration of a drug and incubated at 37°C for 7 days. Then, 10-fold serially diluted cultures were spread onto Middlebrook 7H10 (BD, USA) agar plates. After 3 weeks of incubation, colonies were counted.

### Microbial cell viability assay

A luminescent viability assay kit (G8231, Promega, USA) was used to quantitively analyze changes in the viability of mycobacteria caused by drugs. It was performed according to the manufacturer’s instructions. Briefly, *M*. *tuberculosis* (1.6 × 10^5^ CFU/ml) was inoculated into 7H9 broth (BD, USA) containing 10% ADC (BD, USA) and 0.2% glycerol (Samchun, Korea). Then, 200 μl aliquots containing various concentrations of drug were dispensed into 96-well plates. After wrapping in foil, plates were incubated at 37°C for 7 days. After incubation, mycobacterial cells were completely resuspended, 100 μl of the cell suspension was mixed with 100 μl of BacTiter-Glo reagent (Promega, USA) and then incubated on an orbital shaker at room temperature for 10 minutes. Luminescence as an indicator of viable cells was measured with a multilabel reader (Victor X3, Perkin Elmer, USA).

### Intracellular antimycobacterial activity

Murine Raw 264.7 macrophages (KCLB 40071) were purchased from the Korean Cell Line Bank (KCLB, Korea). Cells were cultured at 37°C in a 5% carbon dioxide incubator using Dulbecco’s Modified Eagle’s Medium (DMEM, Thermo, USA) supplemented with 10% fetal bovine serum (FBS, Sigma, USA). Cell monolayers (1 × 10^6^ cells/ml) grown overnight in 96-well plates were infected with the same volume of *M*. *tuberculosis* at a multiplicity of infection (MOI) of 10:1 and incubated for an additional 2 hours to allow for bacterial uptake. Extracellular *M*. *tuberculosis* was removed through a washing process using DMEM. Cells were then incubated with 200 μl of various concentrations of a drug for 3 days. After incubation, cells were lysed with 0.2% Triton X-100 (Thermo, USA) for 20 minutes at 37°C. Serially diluted lysates were plated onto 7H10 (BD, USA) agar plates to determine the number of viable bacteria. The effect of PP2S (1 μg/ml) on GFP-expressing *M*. *tuberculosis*-infected macrophage cells was analyzed using confocal microscopy as previously reported [27,28].

### Cytotoxicity test

A549 human pulmonary epithelial cells (KCLB 10185), human embryonic kidney (HEK) 293 cells (KCLB 21573), human THP-1 leukemia cells (KCLB 40202), human neuroblastoma SH-SY5Y cells (KCLB 22266), Murine Raw 264.7 macrophages (KCLB 40071), and L929 murine fibroblasts (KCLB 10001) were purchased from the KCLB (Seoul, Korea). Cytotoxicity testing was performed using the MTT assay [29] and the WST-1 cell viability assay [30] as previously described.

### In vivo efficacy in a mouse model of tuberculosis

A tuberculosis infection model was established as previously reported [31] with some modifications. Briefly, 6-week-old BALB/c female mice were purchased from Dooyeol Biotech (Korea). Six mice per group were tested. Mice were infected with *M*. *tuberculosis* H37Rv at a low dose of less than about 50 CFU per mouse through the respiratory tract using a nebulizer (InnoSpire Essence, Philips, the Netherlands). After 4 weeks of infection, 15 and 30 mg/kg body weight of PP1S were orally administered using a zonde once a day, 5 times a week for 4 weeks. Corn oil (Sigma, USA) was used as the vehicle. A high-dose infection model was also established, and the efficacy of PP2S was evaluated. Mice were challenged with a high-dose aerosol of *M*. *tuberculosis* H37Rv (approximately 4 to 5 log CFU per mouse). From 10 days after infection, mice were administered with 30 or 100 mg/kg body weight of the drug dissolved in corn oil 5 days a week for 2 weeks. The initial amount of *M*. *tuberculosis* infection was confirmed through the CFU test by extracting the lungs of 3 simultaneously infected mice the day after infection. After drug administration, *M*. *tuberculosis* in the lungs was also confirmed through the CFU test. For quantitative analysis of CFU of *M*. *tuberculosis* in the lung, 1 side of the lung was placed in a 1.4-mm diameter glass beads tube (Lysing Matrix D, MP Biomedicals, USA) with 1 ml of 0.85% NaCl solution after lung extraction and physically disrupted using a bead-based homogenizer (BeadBug, Benchmark Scientific, USA). Homogenized samples were then diluted and spread onto 7H10 agar plates followed by incubation for 3 weeks in a 37°C incubator. For histopathology analysis, the left lung was fixed with 10% neutral formalin, embedded in paraffin, and processed for histology. Sections were stained with hematoxylin–eosin and Ziehl–Neelsen. All animal studies were approved by the Institutional Animal Care and Use Committee (IACUC) of Soonchunhyang University (approval number: SCH18-0029) according to the Korean Animal Protection Law.

### Acute/subchronic toxicity and genotoxicity toxicity studies

Toxicity studies were conducted at Biotoxtech (Korea) under review and approval from IACUC (170842, 180144, 170889) of Biotoxtech. All animals used in the toxicity test were purchased from OrientBio (Korea).

For acute toxicity study with a single oral administration, 6-week-old Crl:CD SD rats were used. For PP1S, a total of 8 groups (*n* = 4 rats/group) were used (0, 500, 1,000, and 2,000 mg/kg for females and males, respectively). For PP2S, a total of 4 groups (*n* = 5 rats/group) were used (0 and 2,000 mg/kg for females and males, respectively). After administering the drug once by oral gavage at 10 mL/kg body weight, clinical symptoms were observed for 2 weeks, and body weight was measured. Corn oil (Sigma, USA) was used as the vehicle. Control animals were treated only with the same volume of the vehicle.

A subchronic toxicity study was performed for PP2S. Female and male rats were divided into a control group (0 mg/kg/day, *n* = 15), a low-dose group (167 mg/kg/day, *n* = 10), a medium-dose group (500 mg/kg/day, *n* = 10), and a high-dose group (1,500 mg/kg/day, *n* = 15), respectively. Thus, there were a total of 8 groups. The drug was administered by oral gavage at 10 mL/kg body weight daily for 4 weeks. Five animals each in the control and high-dose groups continued their treatments for 2 more weeks. Clinical symptoms were observed, and body weight was measured during the test period for a total of 6 weeks; 0.5% methyl cellulose 1,500 cP (Sigma, USA) in water for injection (JW Pharmaceutical, Korea) was used as a vehicle. Control animals were treated with only the same volume of the vehicle.

Genotoxicity studies for PP2S were performed according to the ICH (International conference on harmonization) harmonized tripartite guideline, S2(R1) [32]. Regarding previous reports, the bacterial reverse mutation test [33–35], chromosomal abnormality test [36,37], and mouse bone marrow micronucleus test [38,39] were performed with slight modifications.

### Species belonging to *Mycobacterium* genus other than tuberculosis species

*Mycobacterium abscessus* KMRC 00136–61038, *Mycobacterium arupense* KMRC 00136–15004, *Mycobacterium aubagnense* KMRC 00136–72001, *Mycobacterium avium* KMRC 00136–41012, *Mycobacterium bolletii* KMRC 00136–52003, *Mycobacterium bovis* NCCP 14790, *Mycobacterium chitae* KMRC 00136–80001, *Mycobacterium colombiense* KMRC 00136–86001, *Mycobacterium conceptionense* KMRC 00136–79001, *Mycobacterium fortuitum* KMRC 00136–60002, *Mycobacterium gilvum* KCTC-19423, *Mycobacterium goodie* KMRC 00136–28003, *Mycobacterium gordonae* KMRC 00136–32003, *Mycobacterium heraklionease* KMRC 00136–81001, *Mycobacterium intracellulare* KMRC 00136–43007, *Mycobacterium kansasii* KMRC 00136–20004, *Mycobacterium kyorinense* KMRC 00136–82002, *Mycobacterium marinum* KMRC 00136–21108, *Mycobacterium marseiliense* KMRC 00136–83001, *Mycobacterium massiliense* KMRC 00136–13017, *Mycobacterium neoaurum* KMRC 00136–18001, *Mycobacterium peregrinum* KMRC 00136–75003, *Mycobacterium phlei* KMRC 00136–19002, *Mycobacterium phocaicum* KMRC 00136–22005, *Mycobacterium smegmatis* KCTC-9108, *Mycobacterium szulgai* KMRC 00136–61005, *Mycobacterium xenopi* KMRC 00136–42003) strains were purchased from the Korean Microorganism Resource Center (KMRC) (Chungbuk, Korea) and the Korean Collection for Type Cultures (KCTC) (Jeonbuk, Korea). MICs of compounds against mycobacteria were determined with the resazurin microtiter assay.

### General antibacterial and antifungal activity

Bacteria (*Acinetobacter baumannii* NCCP 14782, *Citrobacter freundii* NCCP 14766, *Enterobacter aerogenes* NCCP 14761, *Escherichia coli* NCCP 14762, *Escherichia coli* O157 NCCP 14541, *Klebsiella pneumoniae* NCCP 14764, *Proteus mirabilis* NCCP 14763, *Proteus vulgaris* NCCP 14765, *Pseudomonas aeruginosa* NCCP 14781, *Salmonella Enteritidis* NCCP 14771, *Salmonella Paratyphi* A NCCP 14759, *Salmonella Typhimurium* NCCP 16207, *Serratia marcescens* NCCP 14770, *Shigella boydii* NCCP 14745, *Shigella dysenteriae* NCCP 14746, *Shigella flexneri* NCCP 14744, *Shigella sonnei* NCCP 14773, *Bacillus subtilis* NCCP 11101, *Corynebacterium diphtheriae* NCCP 10353, *Staphylococcus aureus* MSSA NCCP 14780, *Staphylococcus aureus* MRSA NCCP 14769, *Staphylococcus epidermidis* NCCP 14768, *Streptococcus pneumoniae* NCCP 14774) and fungi (*Aspergillus fumigatus* NCCP 22454, *Candida albicans* NCCP 32557, *Cryptococcus neoformans* NCCP 32559, *Microsporum canis* NCCP 22454, *Rhizopus oryzae* NCCP 22458, *Saccharomyces cerevisiae* NCCP 32558, *Trichophyton rubrum* NCCP 22456) strains were obtained from the National Culture Collection for Pathogens (NCCP) (Chungbuk, Korea). The MIC of each agent was determined following the Clinical and Laboratory Standards Institute (CLSI).[40] The MIC value was determined as the lowest drug concentration that showed complete inhibition of the visible growth of an organism.

### Analysis of drug-mediated microbiome changes

Six-week-old BALB/c female mice were used for this study (6 mice for each group). Each drug was administered orally at 30 mg/kg once daily for 7 days. Stool was collected, and 16 rRNA gene-based metagenomics analysis was performed. Corn oil was used as the vehicle. The drug-free group received corn oil only. This animal experiment was conducted with the approval of the IACUC of the Soonchunhyang University (approval number: SCH21-0011). Microbiome analysis of collected feces was carried out as previously described [41] with some modifications. Total DNA was extracted from the stool using the QIAamp DNA stool mini kit (Qiagen, Germany) after mechanical disruption using lysing matrix B tubes (MP Biomedicals, USA). V4 region of 16S RNA gene was amplified from extracted DNA with the following primers: forward primer (TCGTCGGCAGCGTCAGATGTGTATAAGAGACAGCCTACGGGNGGCWGCAG) and reverse primer (GTCTCGTGGGCTCGGAGATGTGTATAAGAGACAGGACTACHVGGGTATCTAATCC). PCR was performed using the Kappa HiFi hot start kit (KAPA Biosystems, USA) for 16s gene amplification and library preparation. A dsDNA HS assay kit and Qubit 4 fluorimeter (Invitrogen, USA) were used for DNA quantification. After each step, quality was checked using Agilent High Sensitivity DNA Kit and Agilent Bioanalyzer 2100 (Agilent Technologies, USA). Clean-up was performed using AMPure XP beads (Beckman Coulter, UK). The metagenomic library was prepared using a Nextera XT DNA Library Prep Kit (Illumina, USA) and loaded into an iSeq-100 reagent cartridge (Illumina, USA) together with a PhiX Control library (Illumina, USA). A 300-bp paired-end sequencing was performed on an iSeq-100 platform (Illumina, USA). Sequencing data were analyzed using the EzBioCloud server (Chunlab, Korea).

### Whole-genome sequencing of drug-resistant strain

Spontaneous mutants resistant to PPs were selected by growing *M*. *tuberculosis* H37Rv on 7H10 agar plates containing drugs. Extraction of genomic DNA from *M*. *tuberculosis* was performed using QIAamp DNA Mini Kit (Qiagen, Germany) after mechanical disruption using lysing matrix B tubes (MP Biomedicals, USA). Whole-genome sequencing was performed by Macrogen (order number: 1512KPB-0017, 1702AHF-0023, Korea) using HiSeq X ten (Illumina, USA) and PacBio RS II (Pacific Biosciences, USA). Whole genomes of PPs-resistant strains were compared with whole-genome data of *M*. *tuberculosis* H37Rv using a comparative genome analysis method.

### Point mutagenesis in *M*. *tuberculosis*

The point mutation technique based on single-stranded DNA recombination was applied as previously reported and performed with minor modifications [42]. First, as in the previous report, an electrocompetent *M*. *tuberculosis* H37Rv strain was prepared, and a plasmid pJV62 (plasmid#26910, Addgene, USA) was introduced by an electroporation method [43]. *M*. *tuberculosis* was cultured with an OD600nm value of 0.8 to 1.0 and washed 3 times in 10% glycerol. Electrocompetent cells (200 μl) containing 100 ng of plasmid DNA were electroporated at 1,000 Ω, 2.5 kV, and 25 μF using Gene Pulser II (Bio-Rad, USA). Cells were recovered at 37°C in 1 ml of 7H9 broth containing ADC and 0.05% Tween 80. The introduction of the pJV62 plasmid was confirmed using a primer set (Forward: TCCGGTCTACTTCTACGCGA, Reverse: AATTCCCTGATCTCGTCGGC) for Che9c gene 61 at positions 4574 bp to 5635 bp of the vector. The oligonucleotides of ssDNA were then electroporated. *M*. *tuberculosis* containing pJV62 was grown to the mid-log stage in 7H9 broth supplemented with ADC, 0.05% Tween 80, pantothenate (100 mg/ml), and kanamycin (20 mg/ml). Incubated with 0.2% acetamide at 37°C for 24 hours to induce gene expression as previously reported [44], electrocompetent cells were prepared in the same manner as above. ssDNA (100 ng) was electroporated and then recovered in 7H9 medium supplemented with ADC and Tween 80 for 3 days and plated on 7H10 agar containing ADC and kanamycin. A lead primer (CGGCACCAACGGCTCCGGCGGCGCCGGCGGCACCGGCGGACAAGGCGGCG CCGGGGGTGCTGGCGGGGCCG) and a lag primer (CGGCCCCGCCA GCACCCCCGGCGCCGCCTTGTCCGCCGGTGCCGCCGGCGCCGCCGGAGCCGTTGGTGCCG) were used for mutation at position 2261 of Rv3514.

## Supporting information

S1 Scheme General scheme for the synthesis of PPs. (TIF)

S2 Scheme General scheme for the synthesis of DPG analogs. (TIF)

S1 Supporting Chemistry Schemes Supporting chemistry schemes, synthesis, and characterization. (DOCX)

S1 Table Structures, in vitro antitubercular activities, and cytotoxicities of DPGA. (DOCX)

S2 Table In vitro antitubercular activities of PPs. (DOCX)

S3 Table Summary of histopathological findings. (DOCX)

S4 Table Organ weights during the subchronic oral toxicity test. (DOCX)

S5 Table Results for the number of revertant colonies per plate in the presence or absence of S9 mix. (DOCX)

S6 Table Chromosomal aberration test results. (DOCX)

S7 Table Bone marrow micronucleus test results. (DOCX)

S8 Table Summary of deaths of mice during the acute oral toxicity test. (DOCX)

S9 Table In vitro antimycobacterial activities against strains of *M*. *bovis* BCG. (DOCX)

S1 Fig Circular dichroism spectra of PPs. Data underlying this Figure can be found in S1 Data. (TIF)

S2 Fig Intracellular killing activity of PP2S against GFP-expressing *M*. *tuberculosis*. (TIF)

S3 Fig Images of Ziehl–Neelsen staining in a high-dose infection model. (TIF)

S4 Fig Plasma concentration-time profiles after single oral administration to female rats. Data underlying this Figure can be found in S1 Data. (TIF)

S5 Fig PP2S-resistant *M*. *tuberculosis* strain. (TIF)

S1 Data Excel spreadsheet containing, in separate sheets, the underlying numerical data and statistical analysis for Figs 1C, 2A–2D, 3AB, 4AC, 5A–5D, 6A–6D, 7C, S1 and S4. (XLSX)

## Abbreviations

BCG: bacillus Calmette–Guérin
BLAST: Basic Local Alignment Search Tool
CLSI: Clinical and Laboratory Standards Institute
DPG: deoxypergularinine
EMB: ethambutol
GFP: green fluorescent protein
HE: hematoxylin–eosin
HPLC: high-performance liquid chromatography
INH: isoniazid
MDR: multidrug-resistant
MOI: multiplicity of infection
MRSA: methicillin-resistant *Staphylococcus aureus*
MSSA: methicillin-susceptible *Staphylococcus aureus*
NCBI: National Center for Biotechnology Information
NTM: nontuberculosis mycobacteria
PERMANOVA: permutational multivariate analysis of variance
PP: methyl (S)-1-((3-alkoxy-6,7-dimethoxyphenanthren-9-yl)methyl)-5-oxopyrrolidine-2-carboxylate
PP1S: (S)-N-[(2,3,6-Trimethoxy-10-phenanthryl)methyl]-pyroglutamic acid methyl ester
PP2S: Methyl (S)-1-((3-butoxy-6,7-dimethoxyphenanthren-9-yl)methyl)-5-oxopyrrolidine-2-carboxylate
PP3S: Methyl (S)-1-{[3-(Benzyloxy)-6,7-dimethoxy-9-phenanthryl]methyl}-5-oxopyrrolidine-2-carboxylate
PP3R: Methyl (R)-1-{[3-(Benzyloxy)-6,7-dimethoxy-9-phenanthryl]methyl}-5-oxopyrrolidine-2-carboxylate
PZA: pyrazinamide
RIF: rifampicin
SAR: structure–activity relationship
SD: Sprague Dawley
STR: streptomycin
TDR: totally drug-resistant
TLC: thin-layer chromatography
VAN: vancomycin
XDR: extensively drug-resistant
