# Supplementary material for: A novel class of antimicrobial drugs selectively targets a Mycobacterium tuberculosis PE-PGRS protein
Source: PLoS Biol. 2022 May 31;20(5):e3001648. doi: 10.1371/journal.pbio.3001648 (PMC9154192; doi:10.1371/journal.pbio.3001648)
Supplement: S1 Supporting Chemistry Schemes — (DOCX) [file pbio.3001648.s003.docx]

**Supplementary Chemistry schemes, synthesis, and characterization**

**(S)-N-[(2,3,6-Trimethoxy-10-phenanthryl)methyl]-pyroglutamic acid methyl ester (PP1S).** According to a previous method [1], 3, 6,​ 7-​trimethoxy​-9-phenanthrenemethanol (3) was synthesized using homoveratric acid (1) and 4-methoxybenzaldehyde (2) as starting materials. (S)-N-[(2, 3, 6-trimethoxy-10-phenanthryl)methyl]-pyroglutamic acid methyl ester (PP1S) was prepared with the published method [2] from compound 3 *in situ*. A solution of PBr_3_ (2.72 g, 10.1 mmol) in CHCl_3_ (10 ml) was added dropwise to a solution of 3,6,​7-​trimethoxy​-9-phenanthrenemethanol (2.0 g, 6.70 mmol) in anhydrous CHCl_3_ (150 ml) under nitrogen at 0 ℃. The solution was then stirred at room temperature for 4 hours and poured over ice. Two layers were then separated. The organic phase was dried over anhydrous Na_2_SO_4_, filtered, and concentrated *in vacuo* to yield a bromo compound as a white solid. The bromo compound was then redissolved in N,N-dimethylacetamide (DMF) (200 ml). After adding (S)-dimethyl 2-aminopentanedioate hydrochloride (1.27 g, 6.00 mmol), the solution was stirred for 20 minutes. After adding K_2_CO_3_ (0.83 g, 6.00 mmol), the mixture was stirred at room temperature overnight. The solution was then concentrated using a rotary evaporator and partitioned between CHCl_3_ and H_2_O. The organic layer was dried over anhydrous Na_2_SO_4_, filtered, and concentrated to obtain a crude product. The crude product was dissolved in MeOH (30 ml) and AcOH (10 ml) and stirred for 3 hours at 45 °C. The solution was then evaporated, and the crude product was purified by flash column chromatography to yield 1.39 g (49 % yield) of PP1S [(S)-N-[(2,3,6-trimethoxy-10- phenanthryl)methyl]-pyroglutamic acid methyl ester] as a white solid. MS (ESI) *m*/*z*: 424 (M^+^+1); ^1^H-NMR (400MHz, DMSO-d_6_): δ 8.05 (2H, d, J = 19.2 Hz), 7.82 (1H, d, J = 8.8 Hz), 7.50 (2H, d, J = 8.4 Hz), 7.20 (1H, dd, J = 8.8 & 2.8 Hz), 5.21 (1H, d, J = 14.8 Hz), 4.37 (1H, d, J = 14.4 Hz), 4.01 (3H, s), 3.98 (3H, s), 3.86 (3H, s), 3.84~3.80 (1H, m, chiral CH), 3.44 (3H, s), 2.39~2.28 (2H, m), 2.21~2.11 (1H, m), 1.90~1.83 (1H, m). The synthesis of PP1R was the same as that of PP1S except that (R)-dimethyl 2-aminopentanedioate hydrochloride was used.

**(R)-N-[(2,3,6-Trimethoxy-10-phenanthryl)methyl]-pyroglutamic acid methyl ester (PP1R).** MS (ESI) *m*/*z*: 424 (M^+^+1); ^1^H-NMR (400MHz, DMSO-d_6_): δ 8.03 (2H, d, J = 19.1 Hz), 7.85 (1H, d, J = 8.6 Hz), 7.49 (2H, d, J = 8.3 Hz), 7.21 (2H, d, J = 8.9 Hz), 5.18 (1H, d, J = 14.9 Hz), 4.36 (1H, d, J = 14.3 Hz), 4.06 (3H, s), 3.92 (3H, s), 3.85 (3H, s), 3.82~3.78 (1H, m, chiral CH), 3.42 (3H, s), 2.38~2.26 (2H, m), 2.23~2.10 (1H, m), 1.92~1.81 (1H, m).

**Methyl (S)-1-((3-butoxy-6,7-dimethoxyphenanthren-9-yl)methyl)-5-oxopyrrolidine-2-carboxylate (PP2S).** Compound PP2S was prepared using the same procedures and materials as PP1S except that homoveratric acid (1) and 4-(n-butyloxy)benzaldehyde were used as starting materials. ^1^H NMR (600 MHz, DMSO-d_6_): δ = 8.07 (d, J = 22.2 Hz, 2H), 7.82 (d, J = 9.0 Hz 1H), 7.51 (d, J = 20.4 Hz, 2H), 7.21 (d, J = 8.4 Hz, 1H), 5.34 (d, J = 14.4 Hz, 1H), 4.37 (d, J = 15.0 Hz, 1H), 4.22 (t, J = 12.3 Hz, 2H), 4.03 (s, 3H), 3.88 (s, 3H), 3.83 (dd, J =3.1, 3.1 Hz, 1H), 3.46 (s, 3H), 2.45-2.40 (m, 1H), 2.38–2.32 (m, 1H), 2.20–2.10 (m, 1H), 1.90– 1.86 (m, 1H), 1.81-1.77 (m, 2H), 1.55-1.49 (m, 2H), 0.98 (t, J = 14.7 Hz, 3H). ^13^C NMR (150 MHz, DMSO-d_6_): δ =174.2, 172.1, 157.8, 149.4, 148.9, 131.0, 129.9, 126.6, 126.2, 125.3, 124.7, 124.4, 116.0, 105.0, 104.7, 67.4, 58.2, 55.9, 55.4, 55.3, 52.0, 43.8, 30.9, 29.1, 22.2, 18.9, 13.8. HRMS (ESI) m/z [M + H]^+^ calculated for C_27_H_32_NO_6_: 466.22; found: 466.2226. The synthesis of PP1R was the same as that of PP1S except that (R)-dimethyl 2-aminopentanedioate hydrochloride was used.

**Methyl (R)-1-((3-butoxy-6,7-dimethoxyphenanthren-9-yl)methyl)-5-oxopyrrolidine-2-carboxylate (PP2R).** ^1^H NMR (600 MHz, DMSO-d_6_): δ = 8.06 (d, J = 22.3 Hz, 2H), 7.81 (d, J = 9.2 Hz 1H), 7.49 (d, J = 20.6 Hz, 2H), 7.24 (dd, J = 8.3, 4.5 Hz, 2H), 5.31 (d, J = 14.2 Hz, 1H), 4.39 (d, J = 15.1 Hz, 1H), 4.21 (t, J = 12.6 Hz, 2H), 4.01 (s, 3H), 3.85 (s, 3H), 3.81 (dd, J =3.1, 3.1 Hz, 1H), 3.45 (s, 3H), 2.43-2.39 (m, 1H), 2.39–2.31 (m, 1H), 2.21–2.12 (m, 1H), 1.91– 1.88 (m, 1H), 1.82-1.76 (m, 2H), 1.57-1.52 (m, 2H), 0.96 (t, J = 14.6 Hz, 3H). HRMS (ESI) m/z [M + H]^+^ calculated for C_27_H_32_NO_6_: 466.22; found: 466.2224.

**Methyl (S)-1-{[3-(Benzyloxy)-6,7-dimethoxy-9-phenanthryl]methyl}-5-oxopyrrolidine-2-carboxylate (PP3S).** Compound PP3S and PP3R were synthesized as previously described [3]. ^1^H NMR (600 MHz, DMSO-d_6_): δ =8.21 (s, 1H), 8.03 (s, 1H), 7.97 (d, J =9.1 Hz, 1H), 7.58 (d, J = 7.4 Hz, 2H), 7.42 (dd, J = 6.2, 4.7 Hz, 4H), 7.35 (t, J = 14.6 Hz, 1H), 7.31 (dd, J = 1.3, 1.4 Hz, 1H), 5.39 (s, 2H), 5.22 (d, J = 14.9 Hz, 1H), 4.39 (d, J =14.9 Hz, 1H), 4.03 (s, 3H), 3.92 (s, 3H), 3.90 (d, J =2.5 Hz, 1H), 3.49 (s, 3H), 2.45–2.39 (m, 1H), 2.38–2.33 (m, 1H), 2.25–2.18 (m, 1H), 1.94– 1.90 (m, 1H). ^13^C NMR (150 MHz, DMSO-d_6_): δ =174.2, 172.2, 156.9, 149.5, 149.3, 137.2, 131.4, 128.5 (2×C), 128.0 (2×C), 127.9, 127.4, 126.4, 125.6, 125.1, 124.0, 123.8, 116.1, 108.5, 106.3, 104.3, 69.6, 58.4, 55.9, 55.5, 52.0, 43.4, 29.1, 22.2. HRMS (ESI) m/z [M + H]^+^ calculated for C_30_H_30_NO_6_: 500.20; found: 500.2057. The synthesis of PP3R was the same as that of PP3S except that (R)-dimethyl 2-aminopentanedioate hydrochloride was used.

**Methyl (R)-1-{[3-(Benzyloxy)-6,7-dimethoxy-9-phenanthryl]methyl}-5-oxopyrrolidine-2-carboxylate (PP3R).** ^1^H NMR (600 MHz, DMSO-d_6_): δ =8.17 (s, 1H), 8.07 (s, 1H), 7.84 (d, J =8.7 Hz, 1H), 7.58 (d, J = 7.4 Hz, 2H), 7.51 (d, J = 18.2 Hz, 2H), 7.43 (t, J = 14.9 Hz, 2H), 7.35 (t, J = 14.5 Hz, 1H), 7.29 (dd, J = 1.4, 1.5 Hz, 1H), 5.36 (s, 2H), 5.23 (d, J = 14.6 Hz, 1H), 4.49 (d, J =14.3 Hz, 1H), 4.03 (s, 3H), 3.88 (s, 3H), 3.84 (dd, J =3.1, 3.2 Hz, 1H), 3.46 (s, 3H), 2.45–2.39 (m, 1H), 2.37–2.32 (m, 1H), 2.20–2.13 (m, 1H), 1.90– 1.86 (m, 1H). ^13^C NMR (150 MHz, DMSO-d_6_): δ =174.2, 172.1, 157.3, 149.3, 148.9, 137.2, 130.9, 129.9, 128.5 (2×C), 128.0 (2×C), 127.9, 126.5, 126.4, 125.3, 124.9, 124.4, 116.3, 105.5, 105.0, 104.6, 70.0, 58.2, 55.9, 55.5, 52.0, 43.8, 29.1, 22.2. HRMS (ESI) m/z [M + H]^+^ calculated for C_30_H_30_NO_6_: 500.20; found: 500.2058.

**(E)​-α-​[4-​(Phenylmethoxy)​phenyl]​methylene]​-​3,​4-​dimethoxybenzeneacetic acid (DPGA2)**. A mixture of 3,4-dimethoxyphenylacetic acid (1, homoveratric acid, 1.96 g, 10 mmol), 4-(benzyloxy)benzaldehyde (2, 2.12 g, 10 mmol), triethylamine (1.67 ml, 12 mmol), and acetic anhydride (1.13 mL, 12 mmol) was heated to reflux for 10 hours. It was then cooled, filtered, and washed with small portions of EtOAc to yield DPGA2 (2.93 g, 75%) as a yellow solid. MS (ESI) *m*/*z*: 391 (M^+^+1); 1H-NMR (400MHz, DMSO-d6): δ 7.68 (1H, s), 7.39~7.35 (5H, m), 7.03 (2H, d, J = 9.2 Hz), 6.96 (1H, d, J = 8.4 Hz), 6.86 (2H, d, J = 8.4 Hz), 6.74 (1H, d, 2.0 Hz), 6.68 (1H, dd, J = 8.0 & 2.0 Hz), 5.06 (2H, s), 3.76 (3H, s), 3.64 (3H, s).

**(αE)-3,4-Di​methoxy-​α-​[​4-​(phenylmethoxy)​phenyl]​methylene]​benzeneacetic acid methyl ester (DPGA3).** Acidic DPGA2 (1.95 g, 5.0 mmol) was resolved in SOCl_2_ (0.44 ml, 6.0 mol) and the mixture was refluxed for 30 minutes. Pyridine (0.48 mL, 6.0 mmol) was added after evaporating the solvent. Then 2.0 mL of MeOH was added dropwise while stirring in an ice bath for 30 minutes. After filtering and washing with small portions of MeOH, DPGA3 (1.76 g, 87 %) was obtained as a pale yellow solid. MS (ESI) *m*/*z*: 405 (M^+^+1); ^1^H-NMR (400MHz, DMSO-d_6_): δ 7.68 (1H, s), 7.41~7.35 (5H, m), 7.03 (2H, d, J = 9.2 Hz), 6.96 (1H, d, J = 8.4 Hz), 6.86 (2H, d, J = 8.4 Hz), 6.74 (1H, d, 2.0 Hz), 6.68 (1H, dd, J = 8.0 & 2.0 Hz), 5.06 (2H, s), 3.76 (3H, s), 3.67 (3H, s), 3.64 (3H, s).

**3-​Hydroxy-​6,​7-​dimethoxy-9-phenanthrenecarboxyl​ic acid methyl ester (DPGA4).** To a solution of DPGA3 (1.01 g, 2.5 mmol) in CH_2_Cl_2_ (20 ml), anhydrous FeCl_3_ (1.5 g, 9.3 mmol) in one portion was added on an ice bath. After stirring for 12 hours at room temperature, the reaction was quenched with saturated aqueous NaHCO_3_. The organic layer was collected after filtration and partitioning. The organic layer was then dried with anhydrous Na_2_SO_4_, evaporated, and recrystallized with MeOH to yield DPGA4 (0.24 g, 31%). MS (ESI) *m*/*z*: 313 (M^+^+1); ^1^H-NMR (400 MHz, DMSO-d_6_): δ 8.45 (1H, s), 8.40 (1H, m), 7.96~7.92 (2H, m), 7.28 (1H, d, J = 3.2 Hz), 7.15 (2H, d, J = 8.8 Hz), 3.98 (3H, s), 3.90 (3H, s), 3.89 (3H, s).

**3-Isobutoxy-​6,​7-​dimethoxy-9-phenanthrenecarboxyl​ic acid methyl ester (DPGA5).** A mixture of DPGA4 (0.08 g, 0.26 mmol), K_2_CO_3_ (53 mg, 0.38 mmol), 1-iodo-2-methylpropane (70 mg, 44 μl, 0.38 mmol), and acetone (10 ml) was refluxed for 24 hours, cooled, filtered, concentrated, and purified by chromatography on silica gel using hexane-ethylacetate as an eluent to yield 42 mg (44 % yield) of DPGA5 as a colorless solid. MS (ESI) *m*/*z*: 369 (M^+^+1); ^1^H-NMR (400MHz, DMSO-d_6_): δ 8.43 (2H, d, J = 2.8 Hz), 8.11~8.01 (3H, m), 7.29 (1H, dd, J = 8.8 & 2.4Hz), 4.04 (2H, d, J = 6.8 Hz), 4.03 (3H, s), 3.93 (3H, s), 3.90 (3H, s), 2.13~2.08 (1H, m), 1.05 (6H, d, J = 7.2 Hz).

**3-Cyclohexyloxy-​6,​7-​dimethoxy-9-phenanthrenecarboxyl​ic acid methyl ester (DPGA6).** DPGA6 was prepared by a method analogous to that used for DPGA5. A mixture of DPGA4 (0.08 g, 0.26 mmol), K_2_CO_3_ (53 mg, 0.38 mmol), iodocyclohexane (80 mg, 49 μl, 0.38 mmol), and acetone (10 ml) was refluxed for 24 hours, cooled, filtered, concentrated, and purified by chromatography on a silica gel using hexane-ethylacetate as an eluent to yield 35 mg (34 % yield) of DPGA6 as a colorless solid. MS (ESI) *m*/*z*: 399 (M^+^+1); ^1^H-NMR (400MHz, DMSO-d_6_): δ 8.44 (2H, d, J = 2.0 Hz), 8.10~8.02 (3H, m), 7.29 (1H, d, J = 8.8 Hz), 4.08~4.03 (1H, m), 3.94 (3H, s), 3.93 (3H, s), 3.90 (3H, s), 2.08~1.85 (4H, m), 1.87~1.73 (4H, m), 1.55~1.47 (2H, m).

**3-Ethoxy-​6,​7-​dimethoxy-9-phenanthrenecarboxyl​ic acid methyl ester (DPGA7).** DPGA7 was prepared by a method analogous to that used for DPGA5. A mixture of DPGA4 (0.08 g, 0.26 mmol), K_2_CO_3_ (53 mg, 0.38 mmol), iodoethane (59 mg, 30 μl, 0.38 mmol), and acetone (10 ml) was refluxed for 24 hours, cooled, filtered, concentrated, and purified by chromatography on a silica gel using hexane-ethylacetate as an eluent to yield 23 mg (26 % yield) of DPGA7 as a colorless solid. MS (ESI) *m*/*z*: 341 (M^+^+1); ^1^H-NMR (400 MHz, DMSO-d_6_): δ 8.43 (2H, d, J = 2.0 Hz), 8.12 (2H, d, J = 11.2 Hz), 7.28 (2H, d, J = 7.6 Hz), 4.33~4.06 (2H, m), 4.02 (3H, s), 3.93 (3H, s), 3.90 (3H, s), 2.08~1.85 (4H, m), 1.44 (3H, d, J = 7.0 Hz).

**(E)​-α-​[4-​Butoxy​phenyl]​methylene]​-​3,​4-​dimethoxybenzeneacetic acid (DPGA8).** A mixture of 3,4-dimethoxyphenylacetic acid (1, homoveratric acid, 4.85 ml, 28 mmol), 4-butoxybenzaldehyde (3, 6.59 g, 33.6 mmol), triethylamine (5.80 ml, 42 mmol), and acetic anhydride (11.12 ml, 33.6 mmol) was heated to reflux for 10 hours. It was then cooled, filtered, and washed with small portions of EtOAc to yield DPGA8 (7.78 g, 78 %) as a yellow solid. MS (ESI) *m*/*z*: 357 (M^+^+1); ^1^H-NMR (400MHz, DMSO-d_6_): δ 7.57 (1H, broad s), 6.98 (2H, d, J = 8.8 Hz), 6.91 (1H, d, J = 8.8 Hz), 6.74 (2H, d, J = 8.8 Hz), 6.70 (1H, broad s), 6.63 (1H, d, J = 8.4 Hz), 3.89 (2H, t, J = 6.4 Hz), 3.75 (3H, s), 3.63 (3H, s), 1.62 (2H, m), 1.36 (2H, m), 0.88 (3H, t, J = 7.2 Hz).

**(αE)-3,4-Di​methoxy-​α-​[​4-​butoxy​phenyl]​methylene]​benzeneacetic acid methyl ester (DPGA9).** Acidic DPGA8 (5.06 g, 14.2 mmol) was resolved in SOCl_2_ (3.94 ml, 53.96 mol). The reaction mixture was then refluxed for 30 minutes. Pyridine (4.26 ml, 53.96 mmol) was added after evaporating the solvent. Then 10.0 mL of MeOH was added dropwise while stirring on an ice bath for 30 minutes. After filtration and washing with small portions of MeOH, product DPGA9 (4.79 g, 91 %) was obtained as a pale yellow solid. MS (ESI) *m*/*z*: 371 (M^+^+1); ^1^H-NMR (400 MHz, DMSO-d_6_): δ 7.68 (1H, broad s), 7.03 (2H, d, J = 8.8 Hz), 6.96 (1H, d, J = 8.0 Hz), 6.77 (2H, d, J = 8.8 Hz), 6.74 (1H, d, J = 2.0 Hz), 6.68 (1H, dd, J = 8.0 & 2.0 Hz), 3.91 (2H, t, J = 6.4 Hz), 3.77 (3H, s), 3.67 (3H, s), 3.64 (3H, s), 1.63 (2H, m), 1.36 (2H, m), 0.88 (3H, t, J = 7.6 Hz).

**3-Butoxy-​6,​7-​dimethoxy-9-phenanthrenecarboxyl​ic acid methyl ester (DPGA10).** Anhydrous FeCl_3_ (1.96 g, 12.1 mmol) was added to a solution of DPGA9 (1.21 g, 3.27 mmol) in CH_2_Cl_2_ (30 ml) in one portion on an ice bath. After stirring for 12 hours at room temperature, the reaction was quenched with saturated aqueous NaHCO_3_, and the organic layer was collected after filtration and partitioning. The organic layer was then dried with anhydrous Na_2_SO_4_, evaporated, and recrystallized with MeOH to yield DPGA10 (0.52 g, 43%). MS (ESI) *m*/*z*: 369 (M^+^+1). ^1^H-NMR (400M Hz, DMSO-d_6_): δ 8.44 (2H, d, J = 3.2 Hz), 8.11 (2H, d, J = 11.2 Hz), 8.03 (1H, s), 7.29 (1H, d, J = 8.4 Hz), 4.27 (2H, t, J = 6.4 Hz), 4.05 (3H, s), 3.94 (3H, s), 3.91 (3H, s), 1.80 (2H, m), 1.54 (2H, m), 0.98 (3H, t, J = 7.6 Hz).

**6,​7-​Dimethoxy-​3-butoxy​-9-phenanthrenemethanol​ (DPGA11).** A solution of DPGA10 (1.30 g, 3.53 mmol) in THF (10 ml) was added to a solution of LiAlH_4_ (1.0 M in THF, 10.6 mL, 10.6 mmol) at 0 ℃. After stirring for 30 minutes at 20 ℃, the reaction was terminated with THF/H_2_O (1:1, 10 ml). The mixture was filtered, dried, and then evaporated. The resulting white solid was recrystallized with MeOH to yield DPGA11 (1.10 g, 88 %) as white crystals. MS (ESI) *m*/*z*: 341 (M^+^+1); ^1^H-NMR (400 MHz, DMSO-d_6_): δ 8.08 (1H, broad s), 8.04 (1H, d, J = 3.2 Hz), 7.82 (1H, d, J = 8.8 Hz), 7.64 (1H, s), 7.45 (1H, s), 7.20 (1H, dd, J = 8.4 & 3.2 Hz), 5.30 (1H, broad s, -OH), 4.92 (2H, d, J = 4.8 Hz, -CH_2_-OH), 4.21 (2H, t, J = 6.8 Hz), 4.02 (3H, s), 3.92 (3H, s), 1.79 (2H, m), 1.53 (2H, m), 0.98 (3H, t, J = 7.2 Hz).

**References**

1. Lee YZ, Yang CW, Hsu HY, Qiu YQ, Yeh TK, Chang HY, et al. Synthesis and biological evaluation of tylophorine-derived dibenzoquinolines as orally active agents: exploration of the role of tylophorine e ring on biological activity. J Med Chem. 2012;55(23):10363-77. doi: 10.1021/jm300705j. PubMed PMID: 23167614.

2. Wang Z, Wang L, Ma S, Liu Y, Wang L, Wang Q. Design, synthesis, antiviral activity, and SARs of 14-aminophenanthroindolizidines. J Agric Food Chem. 2012;60(23):5825-31. doi: 10.1021/jf3013376. PubMed PMID: 22662864.

3. Yu P, Lv H, Li C, Ren J, Ma S, Xu S, et al. Stereospecific Synthesis and Biological Evaluation of Monodesmethyl Metabolites of (+)-13a-(S)-Deoxytylophorinine as Potential Antitumor Agents. SYNTHESIS. 2012:3757–64.
