# Supplementary material for: A novel class of antimicrobial drugs selectively targets a Mycobacterium tuberculosis PE-PGRS protein
Source: PLoS Biol. 2022 May 31;20(5):e3001648. doi: 10.1371/journal.pbio.3001648 (PMC9154192; doi:10.1371/journal.pbio.3001648)
Supplement: S1 Table — (DOCX) [file pbio.3001648.s004.docx]

**Table S1** Structures, *in vitro* anti-tubercular activities, and cytotoxicities of DPG analogues

| Compound  no. | Structure | MIC (μg/ml)^a^ |  | CC_50_ (μg/ml)^b^ |  | SI (CC_50_/MIC)^c^ |
| --- | --- | --- | --- | --- | --- | --- |
|  |  | H37Ra | H37Rv | L929 | RAW |  |
| DPGA1  (PP1S) | 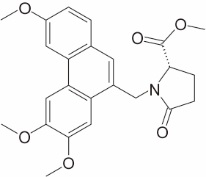 | 1.6 | 1.6 | >100 | >100 | >62.5 |
| DPGA2 | 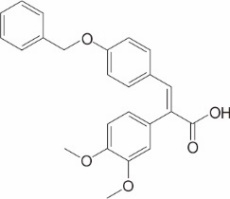 | 100 | >100 | >100 | >100 | ND^d^ |
| DPGA3 | 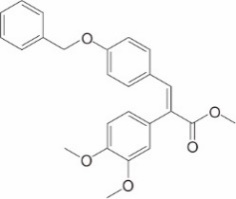 | 3.1 | 3.1 | >100 | >100 | >32.2 |
| DPGA4 | 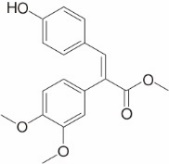 | >100 | >100 | 34.9 | 37.5 | <0.3 |
| DPGA5 | 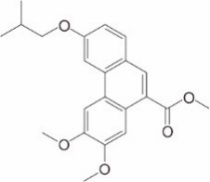 | >100 | >100 | 40.3 | >100 | <0.4 |
| DPGA6 | 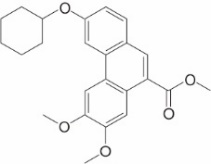 | >100 | >100 | >100 | >100 | ND |
| DPGA7 | 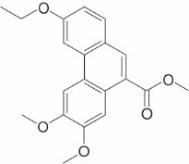 | >100 | >100 | >100 | >100 | ND |
| DPGA8 | 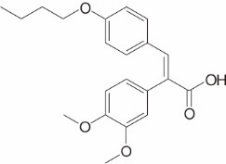 | >100 | >100 | 89.5 | >100 | <0.9 |
| DPGA9 | 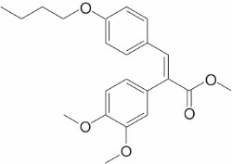 | 25 | 25 | >100 | >100 | >4.0 |
| DPGA10 | 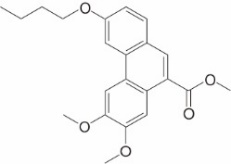 | >100 | >100 | 21.5 | >100 | <0.2 |
| DPGA11 | 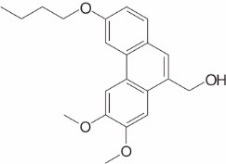 | >100 | >100 | 73.7 | >100 | <0.7 |

^a^Determined by resazurin microtiter assay against *M. tuberculosis* strains H37Ra and H37Rv.

^b^Determined by MTT assay.

^c^SI refers to the ratio of CC_50_ for L929 fibroblasts to the MIC for strain H37Rv (SI = CC_50_/MIC).

^d^ND, not determined.
