# Supplementary material for: A novel class of antimicrobial drugs selectively targets a Mycobacterium tuberculosis PE-PGRS protein
Source: PLoS Biol. 2022 May 31;20(5):e3001648. doi: 10.1371/journal.pbio.3001648 (PMC9154192; doi:10.1371/journal.pbio.3001648)
Supplement: S2 Table — (DOCX) [file pbio.3001648.s005.docx]

**Table S2** *In vitro* anti-tubercular activities of PPs

|  | MIC (μg/ml)^a^ |  |
| --- | --- | --- |
| PPs | H37Ra | H37Rv |
| PP1S | 1.6 | 1.6 |
| PP2S | 0.4 | 0.4 |
| PP3S | 1.6 | 1.6 |
| PP1R | >100 | >100 |
| PP2R | >100 | >100 |
| PP3R | >100 | >100 |

^a^Determined by resazurin microtiter assay against *M. tuberculosis* H37Ra and H37Rv.
