# Supplementary material for: A novel class of antimicrobial drugs selectively targets a Mycobacterium tuberculosis PE-PGRS protein
Source: PLoS Biol. 2022 May 31;20(5):e3001648. doi: 10.1371/journal.pbio.3001648 (PMC9154192; doi:10.1371/journal.pbio.3001648)
Supplement: S3 Table — (DOCX) [file pbio.3001648.s006.docx]

**Table S3** Summary of histopathological findings.

|  |  |  | Dosing period | | | | | | | | Recovery period | | | |
| --- | --- | --- | --- | --- | --- | --- | --- | --- | --- | --- | --- | --- | --- | --- |
|  |  | Sex | Male |  |  |  | Female |  |  |  | Male |  | Female |  |
|  |  | Group | G1 | G2 | G3 | G4 | G1 | G2 | G3 | G4 | G1 | G4 | G1 | G4 |
|  |  | Dose (mg/kg) | 0 | 167 | 500 | 1,500 | 0 | 167 | 500 | 1,500 | 0 | 1,500 | 0 | 1,500 |
| Organ | Findings | No. of animals | 10 | 10 | 10 | 10 | 10 | 10 | 10 | 10 | 5 | 5 | 5 | 5 |
| Adrenal gland | Vacuolation, cortical, diffuse | Grade 1 | 3 | 0 | 0 | 3 | 0 | 0 | 0 | 0 | 2 | 3 | 0 | 0 |
|  |  | Grade 2 | 2 | 0 | 0 | 1 | 0 | 0 | 0 | 0 | 0 | 0 | 0 | 0 |
| Brain | - | - | - | - | - | - | - | - | - | - | - | - | - | - |
| Cecum | - | - | - | - | - | - | - | - | - | - | - | - | - | - |
| Colon | - | - | - | - | - | - | - | - | - | - | - | - | - | - |
| Duodenum | - | - | - | - | - | - | - | - | - | - | - | - | - | - |
| Epididymis | Infiltrate, inflammatory cell, interstitum | Grade 1 | 0 | 0 | 0 | 1 | 0 | 0 | 0 | 0 | 0 | 0 | 0 | 0 |
| Exophagus | - | - | - | - | - | - | - | - | - | - | - | - | - | - |
| Eye | Dysplasia, retina, focal | Grade 1 | 0 | 0 | 0 | 0 | 0 | 0 | 0 | 1 | 0 | 0 | 0 | 0 |
| Femur including bone marrow | - | - | - | - | - | - | - | - | - | - | - | - | - | - |
| Harderian gland | Infiltrate, inflammatory cell, focal | Grade 1 | 0 | 0 | 0 | 1 | 0 | 0 | 0 | 0 | 0 | 0 | 0 | 0 |
| Heart | - | - | - | - | - | - | - | - | - | - | - | - | - | - |
| Ileum | - | - | - | - | - | - | - | - | - | - | - | - | - | - |
| Jejunum | - | - | - | - | - | - | - | - | - | - | - | - | - | - |
| Kidney | Basophilia, tubule, cortex | Grade 1 | 2 | 0 | 0 | 3 | 2 | 0 | 0 | 1 | 2 | 1 | 0 | 0 |
|  | Cast, hyaline, cortex | Grade 1 | 0 | 0 | 0 | 1 | 0 | 0 | 0 | 0 | 3 | 3 | 0 | 0 |
|  | Infiltrate, inflammatory cell, interstitium | Grade 1 | 9 | 0 | 0 | 7 | 4 | 0 | 0 | 4 | 0 | 0 | 3 | 3 |
|  |  | Grade 2 | 0 | 0 | 0 | 1 | 0 | 0 | 0 | 0 | 0 | 0 | 0 | 0 |
|  | Mineralization, medulla | Grade 1 | 1 | 0 | 0 | 0 | 0 | 0 | 0 | 0 | 0 | 0 | 0 | 0 |
|  | Hydronephrosis, unilateral | Grade 3 | 0 | 0 | 0 | 0 | 0 | 0 | 0 | 1 | 0 | 0 | 0 | 0 |
|  | Mineralization, cortex | Grade 1 | 0 | 0 | 0 | 0 | 1 | 0 | 0 | 0 | 0 | 0 | 0 | 0 |
|  | Mineralization, corticomedullary junction | Grade 1 | 0 | 0 | 0 | 0 | 1 | 0 | 0 | 1 | 0 | 0 | 0 | 0 |
| Liver | Extramedullary hematopoiesis | Grade 1 | 1 | 0 | 0 | 1 | 0 | 0 | 0 | 0 | 0 | 1 | 0 | 0 |
|  | Fatty change, sporadic/periportal | Grade 1 | 3 | 0 | 0 | 4 | 5 | 0 | 0 | 5 | 0 | 2 | 2 | 2 |
|  |  | Grade 2 | 0 | 0 | 0 | 0 | 0 | 0 | 0 | 1 | 0 | 0 | 0 | 0 |
|  | Infiltrate, mononuclear cell, focal | Grade 1 | 6 | 0 | 0 | 4 | 5 | 0 | 0 | 2 | 4 | 3 | 3 | 4 |
|  |  | Grade 2 | 0 | 0 | 0 | 0 | 0 | 0 | 0 | 2 | 0 | 0 | 0 | 0 |
| Lung including bronchi | Alveolar macrophage aggregation, focal | Grade 1 | 1 | 0 | 0 | 0 | 0 | 0 | 0 | 0 | 0 | 0 | 0 | 0 |
|  | Infiltrate, mononuclear cell, alveolar, focal | Grade 1 | 1 | 0 | 0 | 2 | 0 | 0 | 0 | 0 | 1 | 0 | 0 | 1 |
| Mammary gland (inguinal) | - | - | - | - | - | - | - | - | - | - | - | - | - | - |
| Mesenteric lymph node | - | - | - | - | - | - | - | - | - | - | - | - | - | - |
| Optic nerve | - | - | - | - | - | - | - | - | - | - | - | - | - | - |
| Pancreas | - | - | - | - | - | - | - | - | - | - | - | - | - | - |
| Parathyroid gland | - | - | - | - | - | - | - | - | - | - | - | - | - | - |
| Pituitary gland | Cyst, pars nervosa | Presence | 0 | 0 | 0 | 1 | 1 | 0 | 0 | 0 | 1 | 0 | 0 | 0 |
| Prostate | Infiltrate, inflammatory cell, interstitium | Grade 1 | 1 | 0 | 0 | 2 | 0 | 0 | 0 | 0 | 2 | 1 | 0 | 0 |
| Rectum | - | - | - | - | - | - | - | - | - | - | - | - | - | - |
| Salivary gland (parotid) | Focus, hypertrophic, basophilic, focal | Grade 1 | 0 | 0 | 0 | 0 | 0 | 0 | 0 | 1 | 0 | 0 | 0 | 0 |
| Salivary gland (sublingual) | - | - | - | - | - | - | - | - | - | - | - | - | - | - |
| Salivary gland (submandibular) | - | - | - | - | - | - | - | - | - | - | - | - | - | - |
| Seminal vesicle | - | - | - | - | - | - | - | - | - | - | - | - | - | - |
| Skin (inguinal) | - | - | - | - | - | - | - | - | - | - | - | - | - | - |
| Spinal cord (thoracic) | - | - | - | - | - | - | - | - | - | - | - | - | - | - |
| Spleen | Pigmentation | Grade 1 | 0 | 0 | 0 | 0 | 4 | 0 | 0 | 4 | 2 | 3 | 5 | 5 |
| Sternum including bone marrow | - | - | - | - | - | - | - | - | - | - | - | - | - | - |
| Stomach | - | - | - | - | - | - | - | - | - | - | - | - | - | - |
| Submandibular lymph node | Increased, plasma cell | Grade 2 | 1 | 1 | 0 | 0 | 0 | 0 | 0 | 0 | 0 | 0 | 0 | 0 |
| Testis | - | - | - | - | - | - | - | - | - | - | - | - | - | - |
| Thymus | - | - | - | - | - | - | - | - | - | - | - | - | - | - |
| Thyroid gland | Ultimobranchial cyst | Presence | 2 | 0 | 0 | 2 | 3 | 0 | 0 | 4 | 2 | 1 | 4 | 2 |
|  | Ectopic thymus | Presence | 0 | 0 | 0 | 0 | 0 | 0 | 0 | 2 | 0 | 0 | 1 | 1 |
| Tongue | - | - | - | - | - | - | - | - | - | - | - | - | - | - |
| Trachea | - | - | - | - | - | - | - | - | - | - | - | - | - | - |
| Urinary bladder | - | - | - | - | - | - | - | - | - | - | - | - | - | - |
| Uterus | - | - | - | - | - | - | - | - | - | - | - | - | - | - |
| Vagina | - | - | - | - | - | - | - | - | - | - | - | - | - | - |

Histopathological examination was performed while performing a 4-week repeated oral toxicity study for PP2S. Organs and tissues from all animals subjected to autopsy were removed and fixed in 10% neutral buffered formalin. Among them, testes, eyeballs and optic nerve were fixed in Davidson's fixative and then in 10% neutral buffered formalin. For organs/tissues subjected to histopathological examination, specimens were prepared according to the histopathological specimen preparation SOP. For histopathological examination, organs and tissues of all subjects in the control group and the high-dose group, and macroscopically observed subjects in the low-dose group were examined. In all administration groups, no change was observed due to administration of the substance. In addition, all other changes observed in histopathological examination were judged to be spontaneous or accidental. Those not specifically observed for all groups by organ were marked with a 'dash' symbol. Grade 1, minimal; Grade 2, slight; Grade 3, moderate; Grade 4, severe.
