# Supplementary material for: A novel class of antimicrobial drugs selectively targets a Mycobacterium tuberculosis PE-PGRS protein
Source: PLoS Biol. 2022 May 31;20(5):e3001648. doi: 10.1371/journal.pbio.3001648 (PMC9154192; doi:10.1371/journal.pbio.3001648)
Supplement: S4 Table — (DOCX) [file pbio.3001648.s007.docx]

**Table S4** Organ weights during the subchronic oral toxicity test.

|  |  |  | Dosing period | | | | | | | | Recovery period | | | |
| --- | --- | --- | --- | --- | --- | --- | --- | --- | --- | --- | --- | --- | --- | --- |
|  |  | Sex | Male |  |  |  | Female |  |  |  | Male |  | Female |  |
|  |  | Group | G1 | G2 | G3 | G4 | G1 | G2 | G3 | G4 | G1 | G4 | G1 | G4 |
|  |  | Dose (mg/kg) | 0 | 167 | 500 | 1,500 | 0 | 167 | 500 | 1,500 | 0 | 1,500 | 0 | 1,500 |
|  |  | No. of animals | 10 | 10 | 10 | 10 | 10 | 10 | 10 | 10 | 5 | 5 | 5 | 5 |
| Mean | B.W. | Mean | 382.1 | 363.4 | 363.5 | 366.0 | 223.7 | 218.0 | 224.1 | 217.3 | 416.2 | 419.4 | 230.2 | 248.7 |
| absolute |  | S.D. | 20.7 | 22.7 | 19.1 | 29.4 | 16.6 | 19.7 | 15.3 | 8.5 | 8.9 | 31.6 | 15.4 | 11.6 |
| organ | Brain | Mean | 1.86 | 1.79 | 1.74 | 1.81 | 2.03 | 2.02 | 1.87 | 1.86 | 2.03 | 2.02 | 1.87 | 1.86 |
| weights |  | S.D. | 0.12 | 0.07 | 0.09 | 0.08 | 0.11 | 0.08 | 0.08 | 0.07 | 0.11 | 0.08 | 0.08 | 0.07 |
| (g) | Pituitary | Mean | 0.0112 | 0.0109 | 0.0119 | 0.0113 | 0.0143 | 0.0140 | 0.0139 | 0.0139 | 0.0123 | 0.00118 | 0.0131 | 0.0154 |
|  |  | S.D. | 0.0018 | 0.0017 | 0.0012 | 0.0013 | 0.0012 | 0.0022 | 0.0020 | 0.0020 | 0.0013 | 0.0015 | 0.0024 | 0.0029 |
|  | Heart | Mean | 1.32 | 1.33 | 1.31 | 1.33 | 0.87 | 0.87 | 0.89 | 0.84 | 1.42 | 1.36 | 0.87 | 0.91 |
|  |  | S.D. | 0.15 | 0.07 | 0.13 | 0.11 | 0.07 | 0.10 | 0.09 | 0.05 | 0.08 | 0.10 | 0.07 | 0.09 |
|  | Lung | Mean | 1.43 | 1.42 | 1.41 | 1.45 | 1.13 | 1.09 | 1.10 | 1.11 | 1.57 | 1.61 | 1.07 | 1.17 * |
|  |  | S.D. | 0.14 | 0.09 | 0.09 | 0.13 | 0.11 | 0.08 | 0.03 | 0.07 | 0.06 | 0.10 | 0.08 | 0.04 |
|  | Liver | Mean | 11.32 | 10.38 | 10.46 | 10.79 | 6.64 | 6.63 | 6.67 | 6.49 | 11.49 | 11.62 | 6.26 | 7.01 * |
|  |  | S.D. | 1.21 | 0.66 | 1.09 | 1.32 | 0.71 | 0.61 | 0.65 | 0.62 | 0.60 | 1.49 | 0.57 | 0.45 |
|  | Spleen | Mean | 0.81 | 0.69 | 0.69 | 0.74 | 0.47 | 0.50 | 0.47 | 0.48 | 0.77 | 0.72 | 0.51 | 0.54 |
|  |  | S.D. | 0.15 | 0.11 | 0.10 | 0.09 | 0.08 | 0.07 | 0.03 | 0.07 | 0.12 | 0.18 | 0.10 | 0.14 |
|  | Kidney | Mean | 2.66 | 2.59 | 2.67 | 2.63 | 1.71 | 1.77 | 1.70 | 1.72 | 2.89 | 2.73 | 1.73 | 1.73 |
|  |  | S.D. | 0.19 | 0.20 | 0.13 | 0.20 | 0.16 | 0.22 | 0.16 | 0.13 | 0.07 | 0.24 | 0.23 | 0.13 |
|  | Adrenal | Mean | 0.0654 | 0.0619 | 0.0622 | 0.0630 | 0.0754 | 0.0775 | 0.0755 | 0.0730 | 0.0636 | 0.0595 | 0.0752 | 0.0723 |
|  |  | S.D. | 0.0079 | 0.0104 | 0.0081 | 0.0112 | 0.0097 | 0.0074 | 0.0090 | 0.0107 | 0.0073 | 0.0169 | 0.0090 | 0.0058 |
|  | Testis/  Ovary | Mean | 3.43 | 3.22 | 3.32 | 3.35 | 0.0905 | 0.0898 | 0.0846 | 0.0874 | 3.30 | 3.48 | 0.0904 | 0.1012 |
|  |  | S.D. | 0.26 | 0.27 | 0.21 | 0.28 | 0.0118 | 0.0184 | 0.0192 | 0.0150 | 0.34 | 0.31 | 0.0119 | 0.0137 |
|  | Prostate/  Uterus | Mean | 0.47 | 0.40 | 0.49 | 0.45 | 0.56 | 0.52 | 0.53 | 0.47 | 0.59 | 0.59 | 0.65 | 0.54 |
|  |  | S.D. | 0.10 | 0.13 | 0.10 | 0.07 | 0.37 | 0.17 | 0.15 | 0.09 | 0.11 | 0.11 | 0.23 | 0.14 |
| Mean | B.W. | Mean | 382.1 | 363.4 | 363.5 | 366.0 | 223.7 | 218.0 | 224.1 | 217.3 | 416.2 | 419.4 | 230.2 | 248.7 |
| relative |  | S.D. | 20.7 | 22.7 | 19.1 | 29.4 | 16.6 | 19.7 | 15.3 | 8.5 | 8.9 | 31.6 | 15.4 | 11.6 |
| organ | Brain | Mean | 0.53 | 0.54 | 0.55 | 0.54 | 0.83 | 0.83 | 0.78 | 0.84 | 0.49 | 0.48 | 0.85 | 0.75 |
| weights |  | S.D. | 0.04 | 0.03 | 0.04 | 0.03 | 0.06 | 0.08 | 0.07 | 0.05 | 0.03 | 0.03 | 0.05 | 0.04 |
| (g/100g | Pituitary | Mean | 0.0029 | 0.0030 | 0.0033 | 0.0031 | 0.0064 | 0.0065 | 0.0062 | 0.0064 | 0.0029 | 0.0028 | 0.0057 | 0.0062 |
| body |  | S.D. | 0.0006 | 0.0004 | 0.0004 | 0.0004 | 0.0004 | 0.0012 | 0.0008 | 0.0008 | 0.0003 | 0.0003 | 0.0010 | 0.0010 |
| weight) | Heart | Mean | 0.35 | 0.37 | 0.36 | 0.37 | 0.39 | 0.40 | 0.40 | 0.38 | 0.34 | 0.33 | 0.38 | 0.37 |
|  |  | S.D. | 0.04 | 0.02 | 0.04 | 0.03 | 0.02 | 0.03 | 0.03 | 0.01 | 0.02 | 0.02 | 0.03 | 0.02 |
|  | Lung | Mean | 0.37 | 0.39 | 0.39 | 0.40 | 0.50 | 0.50 | 0.49 | 0.51 | 0.38 | 0.38 | 0.47 | 0.48 |
|  |  | S.D. | 0.02 | 0.03 | 0.01 | 0.03 | 0.03 | 0.03 | 0.04 | 0.04 | 0.02 | 0.03 | 0.02 | 0.02 |
|  | Liver | Mean | 2.96 | 2.86 | 2.87 | 2.94 | 2.96 | 3.05 | 2.97 | 2.98 | 2.76 | 2.76 | 2.72 | 2.81 |
|  |  | S.D. | 0.22 | 0.09 | 0.20 | 0.17 | 0.15 | 0.20 | 0.17 | 0.17 | 0.17 | 0.15 | 0.12 | 0.09 |
|  | Spleen | Mean | 0.21 | 0.19 | 0.19 | 0.20 | 0.21 | 0.23 | 0.21 | 0.22 | 0.18 | 0.17 | 0.22 | 0.22 |
|  |  | S.D. | 0.04 | 0.03 | 0.03 | 0.02 | 0.03 | 0.02 | 0.01 | 0.03 | 0.03 | 0.03 | 0.03 | 0.05 |
|  | Kidney | Mean | 0.70 | 0.72 | 0.74 | 0.72 | 0.77 | 0.81 | 0.76 | 0.79 | 0.69 | 0.65 | 0.75 | 0.69 |
|  |  | S.D. | 0.06 | 0.06 | 0.05 | 0.03 | 0.08 | 0.08 | 0.06 | 0.05 | 0.02 | 0.05 | 0.05 | 0.05 |
|  | Adrenal | Mean | 0.0172 | 0.0170 | 0.0171 | 0.0172 | 0.0337 | 0.0357 | 0.0337 | 0.0335 | 0.0153 | 0.0140 | 0.0326 | 0.0291 |
|  |  | S.D. | 0.0022 | 0.0025 | 0.0022 | 0.0030 | 0.0035 | 0.0034 | 0.0034 | 0.0041 | 0.0016 | 0.0029 | 0.0020 | 0.0025 |
|  | Testis/  Ovary | Mean | 0.90 | 0.89 | 0.91 | 0.92 | 0.0407 | 0.0410 | 0.0381 | 0.0402 | 0.79 | 0.83 | 0.0392 | 0.0407 |
|  |  | S.D. | 0.07 | 0.08 | 0.09 | 0.06 | 0.0057 | 0.0066 | 0.0097 | 0.0065 | 0.08 | 0.09 | 0.0033 | 0.0054 |
|  | Prostate/  Uterus | Mean | 0.12 | 0.11 | 0.14 | 0.12 | 0.26 | 0.24 | 0.24 | 0.22 | 0.14 | 0.14 | 0.28 | 0.22 |
|  |  | S.D. | 0.03 | 0.03 | 0.03 | 0.02 | 0.19 | 0.09 | 0.06 | 0.04 | 0.03 | 0.02 | 0.09 | 0.06 |

Organ weights were measured while conducting a 4-week repeated oral toxicity study for PP2S. For all animals, wet weight of the following organs were measured, and the relative organ weight ratio to the fasted body weight was calculated. Organs with left and right sides were weighed together. In all administration groups of male and female sexes of the main test group and the recovery group, the effect of the test substance was not recognized. In addition, changes observed with statistical significance in females of the recovery group were minor changes, which were not observed in the main test group. They were not accompanied by other related changes. Thus, they were judged to have no toxicological significance. Significantly different from control by Dunnett's t-test: **p < 0.05*.
