# Supplementary material for: A novel class of antimicrobial drugs selectively targets a Mycobacterium tuberculosis PE-PGRS protein
Source: PLoS Biol. 2022 May 31;20(5):e3001648. doi: 10.1371/journal.pbio.3001648 (PMC9154192; doi:10.1371/journal.pbio.3001648)
Supplement: S5 Table — (DOCX) [file pbio.3001648.s008.docx]

**Table S5** Results for the number of revertant colonies per plate in the presence or absence of S9 mix.

|  |  | In the absence of S9 mix | | | In the presence of S9 mix | | |
| --- | --- | --- | --- | --- | --- | --- | --- |
|  | Dose level | Individual revertant colony counts | | Mean | Individual revertant colony counts | | Mean |
| Strain | (μg/plate) |  |  |  |  |  |  |
| TA98 | DMSO | 18 | 20 | 19 | 37 | 35 | 36 |
|  | 4.88 | 19 | 17 | 18 | 37 | 36 | 37 |
|  | 19.5 | 18 | 17 | 18 | 37 | 35 | 36 |
|  | 78.1 | 17 | 19 | 18 | 42 | 41 | 42 |
|  | 313 | 16 | 15 | 16 | 34 | 33 | 34 |
|  | 2-NF (5.0) | 705 | 649 | 677 |  |  |  |
|  | 2-AA (1.0) |  |  |  | 402 | 414 | 408 |
| TA100 | 0 | 83 | 80 | 82 | 96 | 92 | 94 |
|  | 4.88 | 77 | 71 | 74 | 116 | 111 | 114 |
|  | 19.5 | 74 | 78 | 76 | 114 | 119 | 117 |
|  | 78.1 | 78 | 85 | 82 | 111 | 107 | 109 |
|  | 313 | 98 | 95 | 97 | 111 | 107 | 109 |
|  | SA (1.5) | 734 | 732 | 733 |  |  |  |
|  | 2-AA (2.0) |  |  |  | 555 | 578 | 567 |
| TA1535 | 0 | 16 | 15 | 16 | 14 | 14 | 14 |
|  | 4.88 | 18 | 19 | 19 | 10 | 12 | 11 |
|  | 19.5 | 17 | 19 | 18 | 14 | 16 | 15 |
|  | 78.1 | 18 | 19 | 19 | 17 | 15 | 16 |
|  | 313 | 15 | 17 | 16 | 16 | 15 | 16 |
|  | SA (1.5) | 588 | 535 | 562 |  |  |  |
|  | 2-AA (3.0) |  |  |  | 96 | 103 | 100 |
| TA1537 | 0 | 9 | 7 | 8 | 12 | 13 | 13 |
|  | 4.88 | 10 | 11 | 11 | 16 | 14 | 15 |
|  | 19.5 | 11 | 9 | 10 | 17 | 16 | 17 |
|  | 78.1 | 11 | 10 | 11 | 18 | 17 | 18 |
|  | 313 | 9 | 8 | 9 | 18 | 19 | 19 |
|  | 9-AA (80.0) | 539 | 571 | 555 |  |  |  |
|  | 2-AA (3.0) |  |  |  | 193 | 184 | 189 |
| WP2*uvrA* | 0 | 112 | 102 | 108 | 150 | 154 | 152 |
| (pKM101) | 4.88 | 92 | 86 | 89 | 149 | 156 | 153 |
|  | 19.5 | 86 | 83 | 85 | 145 | 153 | 149 |
|  | 78.1 | 87 | 94 | 91 | 156 | 167 | 162 |
|  | 313 | 91 | 93 | 92 | 162 | 165 | 164 |
|  | 4-NQO (0.1) |  |  |  |  |  |  |
|  | 2-AA (2.0) | 408 | 342 | 375 | 409 | 430 | 420 |

DMSO, dimethyl sulfoxide; 2-NF, 2-nitrofluorene; 2-AA, 2-aminothracene; SA, sodium azide; 9-AA, 9-aminoacridine; 4-NQO, 4-nitroquinoline N-oxide.
