# Supplementary material for: A novel class of antimicrobial drugs selectively targets a Mycobacterium tuberculosis PE-PGRS protein
Source: PLoS Biol. 2022 May 31;20(5):e3001648. doi: 10.1371/journal.pbio.3001648 (PMC9154192; doi:10.1371/journal.pbio.3001648)
Supplement: S6 Table — (DOCX) [file pbio.3001648.s009.docx]

**Table S6** Chromosomal aberration test results

| S9  mix | Trt-  Rec  (hr) | Dose  (µg/mL) | RPD  (%) | No. of  cells  analyzed | Number of cells with  structural aberrations | | | | | | | | | Number of cells with  numerical aberrations | | | Others^a)^ |
| --- | --- | --- | --- | --- | --- | --- | --- | --- | --- | --- | --- | --- | --- | --- | --- | --- | --- |
|  |  |  |  |  | ctb | csb | cte | cse | frg | gap | | total (%) | | end | pol | total (%) |  |
|  |  |  |  |  |  |  |  |  |  | ctg | csg | gap- | gap+ |  |  |  |  |
| - | 6-18 | DMSO | 100 | 300 | 1 | 0 | 0 | 0 | 0 | 0 | 0 | 1 (0.3) | 1 (0.3) | 0 | 1 | 1 (0.3) | 0 |
|  |  | 62.5 | 84.2 | not observed | | | | | | | | | | | | | |
|  |  | 125 | 78.8 | 300 | 0 | 0 | 0 | 0 | 0 | 0 | 0 | 0 (0.0) | 0 (0.0) | 0 | 1 | 1 (0.3) | 0 |
|  |  | 250 | 74.3 | 300 | 0 | 0 | 1 | 0 | 0 | 0 | 0 | 1 (0.3) | 1 (0.3) | 0 | 0 | 0 (0.0) | 0 |
|  |  | MMC (0.1) | 58.2 | 300 | 19 | 0 | 38 | 2 | 0 | 6 | 0 | 67** (22.3) | 68 (22.7) | 0 | 1 | 0 (0.3) | 0 |
| + | 6-18 | DMSO | 100 | 300 | 1 | 0 | 0 | 0 | 0 | 1 | 0 | 1 (0.3) | 2 (0.7) | 0 | 1 | 1 (0.3) | 0 |
|  |  | 62.5 | 79.4 | 300 | not observed | | | | | | | | | | | | |
|  |  | 125 | 69.4 | 300 | 1 | 0 | 1 | 0 | 1 | 0 | 0 | 3 (1.0) | 3 (1.0) | 0 | 0 | 0 (0.0) | 0 |
|  |  | 250 | 67.4 | 300 | 1 | 0 | 3 | 0 | 0 | 2 | 0 | 4 (1.3) | 6 (2.0) | 0 | 2 | 2 (0.7) | 0 |
|  |  | B[a]P (20) | 62.5 | 300 | 10 | 0 | 86 | 2 | 0 | 1 | 0 | 98** (32.7) | 99 (33.0) | 0 | 0 | 0 (0.0) | 0 |
| - | 24-0 | DMSO | 100 | 300 | 0 | 0 | 1 | 0 | 0 | 0 | 0 | 1 (0.3) | 1 (0.3) | 0 | 1 | 1 (0.3) | 0 |
|  |  | 62.5 | 50.7 | 300 | 1 | 0 | 2 | 0 | 0 | 0 | 0 | 3 (1.0) | 3 (1.0) | 0 | 1 | 1 (0.3) | 0 |
|  |  | 125 | 46.9 | 300 | 0 | 0 | 0 | 0 | 0 | 1 | 0 | 0 (0.0) | 1 (0.3) | 0 | 0 | 0 (0.0) | 0 |
|  |  | 250 | 38.6 | 300 | 1 | 0 | 0 | 0 | 0 | 2 | 0 | 1 (0.3) | 3 (1.0) | 0 | 0 | 0 (0.0) | 0 |
|  |  | MMC (0.1) | 52.9 | 300 | 21 | 0 | 87 | 1 | 0 | 2 | 0 | 106** (35.3) | 107 (35.7) | 0 | 0 | 0 (0.0)) | 0 |

Aberration: ctg, chromatid gap; csg, chromosome gap; ctb, chromatid break; cte, chromatid exchange; csb, chromosome break; cse, chromosome exchange; frg, fragmentation; end, endoreduplication; pol, polyploidy; MMC, mitomycin C; B[a]P, benzo[a]pyrene;

RPD, relative population doubling; Trt-Rec time, treatment-recovery time; Gap-, total number of cells with structural aberrations excluding gap; gap+, total number of cells with structural aberrations including gap.

^a)^: Others were excluded from the number of cells with chromosomal aberrations.

Significant difference from negative control by Fisher's exact test: ***p < 0.01.*

N/C: RPD was not calculated due to cytotoxicity.
