# Supplementary material for: A novel class of antimicrobial drugs selectively targets a Mycobacterium tuberculosis PE-PGRS protein
Source: PLoS Biol. 2022 May 31;20(5):e3001648. doi: 10.1371/journal.pbio.3001648 (PMC9154192; doi:10.1371/journal.pbio.3001648)
Supplement: S7 Table — (DOCX) [file pbio.3001648.s010.docx]

**Table S7** Bone marrow micronucleus test results

| Groups | Dose  (mg/kg) | Route | Hours  after dosing | No. of  mice | Mean ± S.D. | | Mean body weights (g) ± S.D. | |
| --- | --- | --- | --- | --- | --- | --- | --- | --- |
|  |  |  |  |  | % PCE | % MNPCE | 0 day | 1 day |
| Negative  control | 0.5% MC  solution | P.O. | 24 | 5 | 32.3 ± 3.11 | 0.035 ± 0.014 | 36.0 ± 1.30 | 35.8 ± 1.63 |
| Test substance | 500 | P.O. | 24 | 5 | 30.5 ± 2.16 | 0.055 ± 0.037 | 35.9 ± 1.16 | 35.5 ± 1.48 |
|  | 1000 | P.O. | 24 | 5 | 31.8 ± 2.49 | 0.060 ± 0.034 | 35.9 ± 1.07 | 36.1 ± 1.42 |
|  | 2000 | P.O. | 24 | 5 | 33.6 ± 1.67 | 0.055 ± 0.021 | 35.9 ± 1.14 | 34.9 ± 1.63 |
| Positive control | MMC (2) | I.P. | 24 | 5 | 33.4 ± 3.15 | 6.33± 0.639 | 35.8 ± 1.09 | 34.9 ± 0.82 |

0.5% MC solution, 0.5% methylcellulose 1,500 centipoise solution; MMC, mitomycin C; P.O., per os.; I.P., intraperitoneal; S.D., standard deviation; PCE, polychromatic erythrocyte; NCE, normochromic erythrocyte; MNPCE, micronucleated polychromatic erythrocyte test.
