# Supplementary material for: A novel class of antimicrobial drugs selectively targets a Mycobacterium tuberculosis PE-PGRS protein
Source: PLoS Biol. 2022 May 31;20(5):e3001648. doi: 10.1371/journal.pbio.3001648 (PMC9154192; doi:10.1371/journal.pbio.3001648)
Supplement: S9 Table — (DOCX) [file pbio.3001648.s012.docx]

**Table S9** *In vitro* antimycobacterial activities against strains of *M. bovis* BCG

|  |  |  |  |  |  | MIC (µg/ml)^a^ | |
| --- | --- | --- | --- | --- | --- | --- | --- |
|  | Query cover | Per. Ident | BioProject | BioSample | GenBAnk/WGS | PP2S | INH |
| *M. bovis* BCG-Tokyo | 98% | 89.55% | PRJNA313242 | SAMN04517448 | CP014566.1 | 0.8 | 0.2 |
| *M. bovis* BCG-Sweden | 32% | 90.15% | PRJEB8560 | SAMEA3257675 | CUWP00000000.1 | >100 | 0.2 |
| *M. bovis* BCG-Praque | 40% | 96.38% | PRJEB8560 | SAMEA3257673 | CUWM00000000.1 | >100 | 0.2 |
| *M. bovis* BCG-Glaxo | 70% | 90.57% | PRJEB8560 | SAMEA3257668 | CUWJ01000001 | >100 | 0.2 |
| *M. bovis* BCG-Danish | 54% | 99.71% | PRJEB8560 | SAMEA3257666 | CUWH01000001 | >100 | 0.2 |
| *M. bovis* BCG-Connauht | 45% | 96.14% | PRJEB8560 | SAMEA3257665 | CUWF01000001 | >100 | 0.2 |
| *M. bovis* BCG-Birkhaug | 32% | 87.52% | PRJEB8560 | SAMEA3257663 | CUWE01000001 | >100 | 0.2 |

^a^Determined by resazurin microtiter assay. *M. bovis* BCG-Tokyo, *M. bovis* BCG-Sweden, *M. bovis* BCG-Praque, *M. bovis* BCG-Glaxo, *M. bovis* BCG-Danish, *M. bovis* BCG-Connauht, and *M. bovis* BCG-Birkhaug were purchased from the Korean Institute of Tuberculosis (Chungbuk, Korea). NCBI (National Center for Biotechnology Information) Nucleotide BLAST (Basic local alignment search tool) analysis revealed that the sequence of *Rv3514* shared the highest similarity with *M. bovis* BCG-Tokyo.
