# Supplementary figures and images for: A novel class of antimicrobial drugs selectively targets a Mycobacterium tuberculosis PE-PGRS protein
Source: PLoS Biol. 2022 May 31;20(5):e3001648. doi: 10.1371/journal.pbio.3001648 (PMC9154192; doi:10.1371/journal.pbio.3001648)

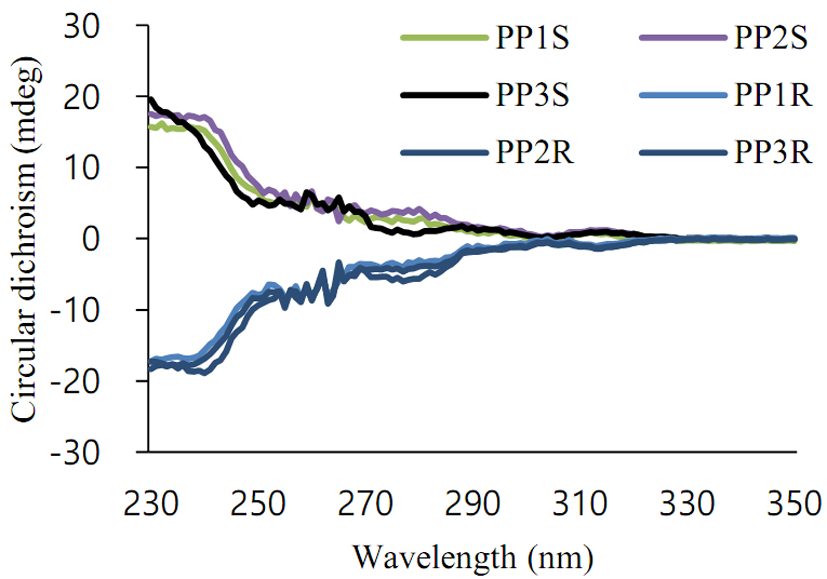

Supplement: S1 Fig — (TIF) [file pbio.3001648.s013.tif]

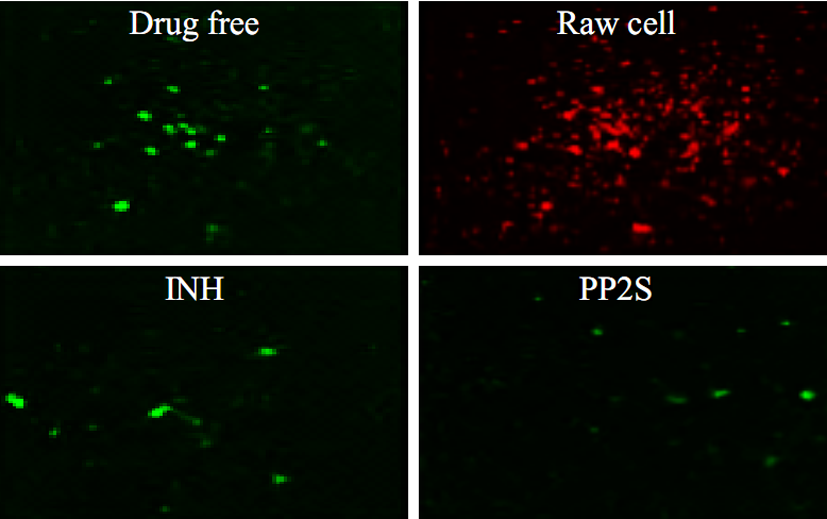

Supplement: S2 Fig — (TIF) [file pbio.3001648.s014.tif]

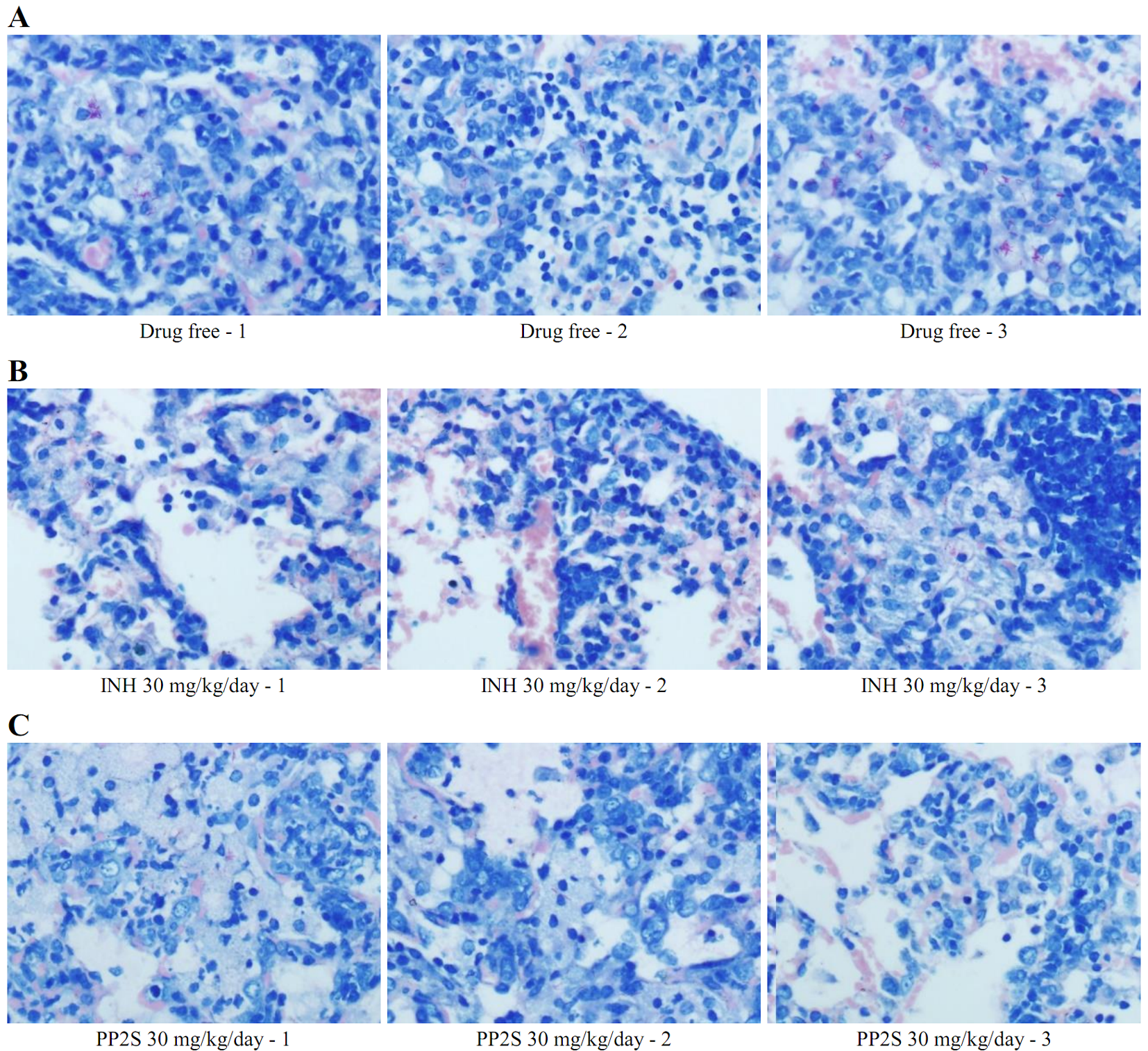

Supplement: S3 Fig — (TIF) [file pbio.3001648.s015.tif]

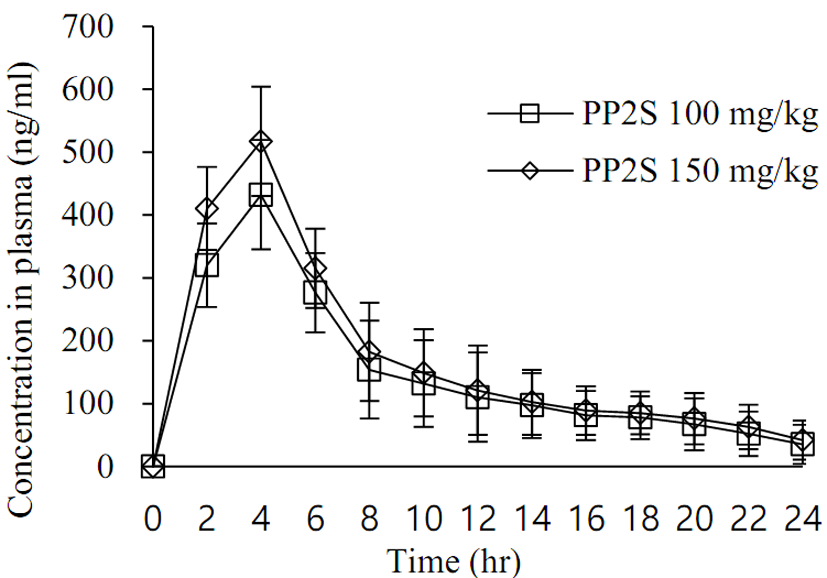

Supplement: S4 Fig — (TIF) [file pbio.3001648.s016.tif]

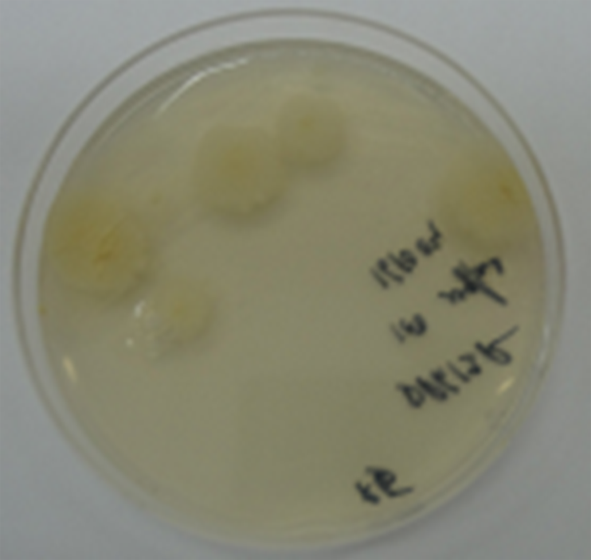

Supplement: S5 Fig — (TIF) [file pbio.3001648.s017.tif]
